# Dynamic shifts in isomiR profiles during parasite maturation of *Fasciola hepatica*

**DOI:** 10.1080/15476286.2025.2538271

**Published:** 2025-07-31

**Authors:** Dayna Sais, Sumaiya Chowdhury, Phuong Thao Nguyen, Krystyna Cwiklinski, Trung Duc Nguyen, Tuan Anh Nguyen, John Dalton, Sheila Donnelly, Nham Tran

**Affiliations:** a School of Biomedical Engineering, Faculty of Engineering and Information Technology Sydney, Sydney, Australia; b The School of Life Sciences, University of Technology Sydney, Sydney, Australia; c Transdisciplinary School, The University of Technology Sydney, Sydney, Australia; d Institute of Infection, Veterinary and Ecological Sciences, University of Liverpool, Liverpool, UK; e Division of Life Science, The Hong Kong University of Science and Technology, Hong Kong, China; f Centre for One Health School of Natural Sciences, Ryan Institute, University of Galway, Galway, Ireland; g School of Biological and Chemical Sciences, University of Galway, Galway, Ireland

**Keywords:** Helminth development, microRNAs, RNA regulation, *Fasciola hepatica*, isomiRs

## Abstract

We investigated the isomiR profiles of the parasitic worm *Fasciola hepatica* across three developmental stages: newly excysted juveniles (NEJ), juveniles (JUV), and adults. Our analysis revealed a distinct shift in isomiR distribution during maturation, with NEJs exhibiting a higher abundance and diversity of isomiRs compared to later stages. Notably, isomiRs were often the dominant miRNA form in NEJs, whereas a transition to canonical miRNAs occurred as the parasite matured. This temporal variation suggests that isomiR expression may be linked to the parasite’s life cycle. We observed that truncated isomiRs were more prevalent, with uracil additions at the 3’end and adenosine at the 5’ end being most common. At least 10% of the miRNA population consisted of 5’ end isomiRs, which have the potential to redirect target interactions towards metabolic and developmental pathways. Furthermore, we show that the cleavage sites in *F. hepatica* primary miRNAs are similar to those found in mammalian cells, and Dicer-mediated cleavage appears to play a significant role in isomiR generation. We believe that the diversification of miRNA sequences through isomiR production is an evolutionary adaptation that enhances the parasite’s ability to tune gene expression during infection and development. This regulatory plasticity may facilitate successful infection and long-term persistence within diverse mammalian hosts. Understanding the roles of isomiRs in parasitic worms could provide new insights into parasite biology and identify potential targets for controlling parasitic infections.

## Introduction

MicroRNAs (miRNAs) are short non-coding RNAs (ncRNAs) that act post-transcriptionally to regulate gene expression through binding of partially complementary mRNAs [1]. They were first discovered in *Caenorhabditis elegans* [2] where they were shown to be temporally expressed across developmental stages of the worm. Progression from the first larval stage L1 to L2 was shown to be under the control of the LIN-14 protein, which in turn was regulated by the lin-4 gene. The unique aspect of this regulation was that Lin-4 gave rise to two small non-coding RNAs with antisense complementarity to the 3`UTR of the LIN-14 transcript. Binding at this region resulted in a decrease in LIN-14 protein expression [2]. These initial observations supported the current paradigm whereby *C. elegans* development is controlled by specific small non-coding RNAs, which are temporally expressed. Since those early studies, it is now understood that miRNAs are ubiquitous in both the plant and animal phyla [3], regulating the expression of a broad range of gene targets to mediate both developmental, and normal, physiological processes [4].

The biogenesis of a typical microRNA occurs over a series of steps, beginning with the initial transcription followed by successive enzymatic cleavage events to generate the canonical miRNA. The nascent transcript is produced in the nucleus by RNA polymerase II. This transcript, known as the primary miRNAs (pri-miRNAs), can be several hundred to several thousand base pairs in size. In the nucleus, it undergoes cleavage by Drosha and its cofactors to generate a precursor miRNA (pre-miRNAs) sequence. This RNA intermediate moves into the cytoplasm via exportin channels for final processing by Dicer to generate the canonical double-stranded 22nt mature miRNAs. These mature miRNAs bear a 5`phosphate and 3`overhang; features that enable the canonical miRNAs to be loaded on Argonaute 2 (Ago2) to become functionalized. In the final processing step, one strand of the miRNA is incorporated into Ago2. This strand acts as a guide for hybridization to complementary sequences on target mRNAs. Target guidance is then determined by the seed region (positions 2 to 8) of the mature miRNA. The outcome of this binding of a miRNA to its target is gene suppression or gene silencing (In-depth reviews for miRNA biogenesis can be found [5,6]).

The processing of the mature miRNAs by Dicer cleavage occurs at specific sequence motifs. While this process is tightly controlled, it is not entirely precise. Through in-depth sequencing, it has become apparent that canonical miRNAs can exist as different length variants known as isomiRs [7]. These isomiRs are grouped into four broad categories according to the type of alteration. Changes in length or sequences at the 5`end are 5`isomiR, and changes in sequence or length at the 3`end are 3`isomiR. Also, polymorphic isomiRs have changes in the internal nucleotide sequence [8,9], and mixed-type isomiRs are a combination of the above types [8,10]. IsomiRs can be further categorised into templated isomiRs, whereby variations in length arise from imprecise cleavage [1,5,11] or from specific exonucleases that shorten the miRNA by removing nucleotides from its ends. In both cases, the resulting isomiRs are classified as templated because their sequences match the canonical sequence. Length differences can also arise from the post-transcriptional addition of a few nucleotides to the 5′ or 3′ end of the mature sequence by nucleotidyl transferases [1,10,12]. These variants are considered non-templated because they include nucleotides that are not present in the corresponding canonical sequence.

The functionality of these isomiRs depends on the modification, albeit most isomiRs are, in fact, loaded onto AGO2. The 3`isomiR are most common and appear to regulate the same targets as their canonical miRNAs as the seed region is essentially the same. In contrast, 5`isomiRs, which result in a shift of the seed region, are potentially redirected to different target genes [13,14]. Although the exact functional role for these isomiRs is not clear, it is now broadly accepted that isomiRs are abundant and often expressed at defined developmental stages and/or in diseased states [8,15].

Similar to the free-living worm *C. elegans*, miRNAs are also temporally expressed by parasitic worms (helminths) and are predicted to control the timing of the expression of genes specifically required for stage-specific development [16–20]. However, to our knowledge, there has been no characterisation of the isomiR profile for any helminth to date. This is important information, as the possession of these miRNA sequence variants would expand/alter the range and depth of gene targets being regulated across the parasite’s development.

To address this gap in knowledge, we have analysed the isomiR expression profile for the intra-mammalian stages of development of the liver fluke, *Fasciola hepatica*. This flatworm is a zoonotic parasite with a unique ability to infect a broad range of mammals, leading to a prevalence on every inhabited continent [21]. *Fasciola hepatica* is excellent model to elucidate the molecular regulation of parasite growth and maturation because it undergoes distinct stages of development that are associated with defined host tissue sites. In addition, the availability of genomes, stage-specific transcriptomics, proteomics and miRNomes have laid a solid foundation on which to dissect the molecular pathways linked to development [22].

Infection with *F. hepatica* occurs following the ingestion of infective metacercaria deposited on vegetation by the intermediate snail hosts. In the small intestine, in response to changes in pH, the newly excysted juveniles (NEJs) emerge and within hours alter their metabolic activities, penetrate the gut wall tissue and migrate across the peritoneal cavity towards the liver [22]. Within the liver parenchyma the parasite develops into juvenile worms displaying digestive and reproductive structures, and undergoes a huge growth phase, doubling in size every few weeks [23]. The parasites finally move into the bile ducts to complete their maturation to adulthood and commence the production of eggs that are carried with the bile juices into the intestine and liberated with the faeces for the life cycle to continue. Accompanying these strict developmental changes are highly regulated changes in gene expression with progressively more genes being expressed with higher fold changes as parasite maturation proceeds [24].

Reflecting these changes in gene expression are stage-specific alterations in miRNA expression, with target prediction suggesting that miRNAs are important in the fine-tuning of metabolism [16,25,26]. Accordingly, it is now important to incorporate a role for isomiRs into these molecular mechanisms of developmental regulation to gain a deeper and more comprehensive understanding of the parasitic gene regulation and to potentially uncover new targets for infection control.

## Methods

### Data set and samples

For this current study we analysed sequencing data sets which we have previously obtained from the NEJ, Juvenile and adult *F. hepatica* (GEO accession: GSE186948) [16]. To obtain these parasite life stages, metacercariae (Italian isolate, Ridgeway Research Ltd, UK) were excysted in vitro and after 24 h culture collected as NEJs [22]. Oral infections of mice and sheep were used to recover 21-day immature flukes (JUV) [27] and adult [28] parasites respectively. Total RNA was extracted from each life stage in triplicate using the miRNeasy mini kit (Qiagen) according to the manufacturers protocol. For each replicate, 1000, 19 and 1 parasites were used for NEJs, JUV and adults respectively. Library preparation of total RNA from samples was performed by ArrayStar using the Small RNA library Prep Set for Illumina and sequenced using Illumina NextSeq 500 (Data File 1).

The quality of the raw and processed sequencing reads was assessed using FastQC v0.11.9 (Data File 1). The Fastq sequencing files were then processed to remove adaptor sequences that were excised and filtered for low quality ( <20 phred scores) sequences, low length sequences ( <16nt), and high length sequences ( > 28nt) using the CutAdapt (v3.4) tool. Mature miRNA sequences from the miRbase *Fasciola* repository (Fheptica_v1, miRbase v21) and other published sources [25,26,29] were aligned to cleared reads using Bowtie2 (v2.4.5).

### IsomiR identification

To identify isomiRs in our RNA sequencing data, we followed the protocol outlined by Panzade et al. [30]. In summary, identical reads were collapsed for unique tag representation into a FASTA file and then converted to a tab-delimited file containing the read sequence tags and their corresponding counts using FASTX-Tool kit (https://github.com/agordon/fastx_toolkit). This tab-delimited file was used as an input file for isomiR identification using the tool isomiR-SEAv1.6 [31]. We ran isomiR-SEAv1.6 with the species code ‘fhe’ based on our custom *Fasciola hepatica* miRNA reference file. The parameters used included minimum tag length (−l 16), seed size (−ss 6), and tag selection threshold (−h 11). The resulting output files were used to retrieve the read counts for the annotated canonical miRNAs and their templated isomiRs across all samples for each life stage. The raw read counts were normalized to reads per million (RPM) of total miRNA counts in each library.

### miRNA biogenesis cleavage prediction and binding motifs

To broadly categorise the potential contribution of Drosha and Dicer processing of these isomiRs, we collected all isomers based on their class type (3`, 5` or both) and their arm of origin (3p or 5p). We categorised each isomiR sequence that was on the 3p arm as 3`DROSHA (if they were 3` altered) or 5`Dicer (if they were 5` altered). If the isomiRs were on the 5p arm, we then categorised them as 5`Drosha (if altered at the 5`end) or 3`Dicer (if they at 3` alterations). For each group, we then calculated the unique tag number and read the abundance across the life stages.

### Pri-miRNA structure analysis

180 pri-miRNA sequences were extracted from the *Fasciola hepatica* genome (PREJEB25283) by extending 20 nt on either side from the pre-miRNA sequences [25,26,29]. Structures of pri-miRNAs were predicted using the RNAfold [32]. The 56 pri-miRNAs with multiple loops were excluded. We estimated the lower stem length and Microprocessor motif in the remaining 124 pri-miRNAs.

We assigned the basal junctions of pri-miRNAs using ssRNA region with more than 6 mismatches from position −3 to −20 on 5p-strands of pri-miRNAs. We identified the lower stem length of a pri-miRNA by counting the number of base pairs from the 5p-end of its pre-miRNA to its basal junction. The DRES motif was scanned from positions −10 to + 4 on the 5p-strands of pri-miRNAs. The UG, midBMW and UGU motifs were searched within positions −14 and −12, +10 and + 12, and + 21 and + 26, on the 5p-strands of pri-miRNAs, respectively. The CNNC motif was searched from positions −16 to −21 on the 3p-strands of pri-miRNAs. The mGHG motif was searched for on the 5p- and 3p-strands of pri-miRNAs from positions −7 to −5 and −5 to −3, respectively. The mGHG scores were collected from the previous report [33].

### IsomiR target prediction

Target prediction was performed on those isomiRs with an alteration at the 5`end and with >5000 RPM. Seed sequences found at the position 2–8 position were extracted for miRNAs and their corresponding 5` isomiRs. *Fasciola* 3`UTR sequences were downloaded from ENSEMBL Biomart from the *Fasciola hepatica* genome (PREJEB25283). We then predicted miRNA:mRNA pairs using TargetScan (http://www.targetscan.org) and miRander (http://www.microrna.org/). miRNA:mRNA pairs were filtered for target score > 155 and minimum free energy (MFE) < −20 (Energy-Kcal/Mol) in miRanda, and a seed sequence match of 7mer-m8 site in TargetScan. The genes predicted to be targeted by the miRNAs were annotated based on the functional annotation of the gene models by Cwiklinski et al. [24].

Gene ontology analysis was performed on predicted miRNA targets using G:profiler (https://biit.cs.ut.ee/gprofiler/gost), and those statistically significant enriched terms were visualized using Revigo (http://revigo.irb.hr/) r-scripts for the scatterplot.

## Results

### Fasciola hepatica *has a distinct shift in isomiR distribution across the developmental life stages*

**Figure 1.**
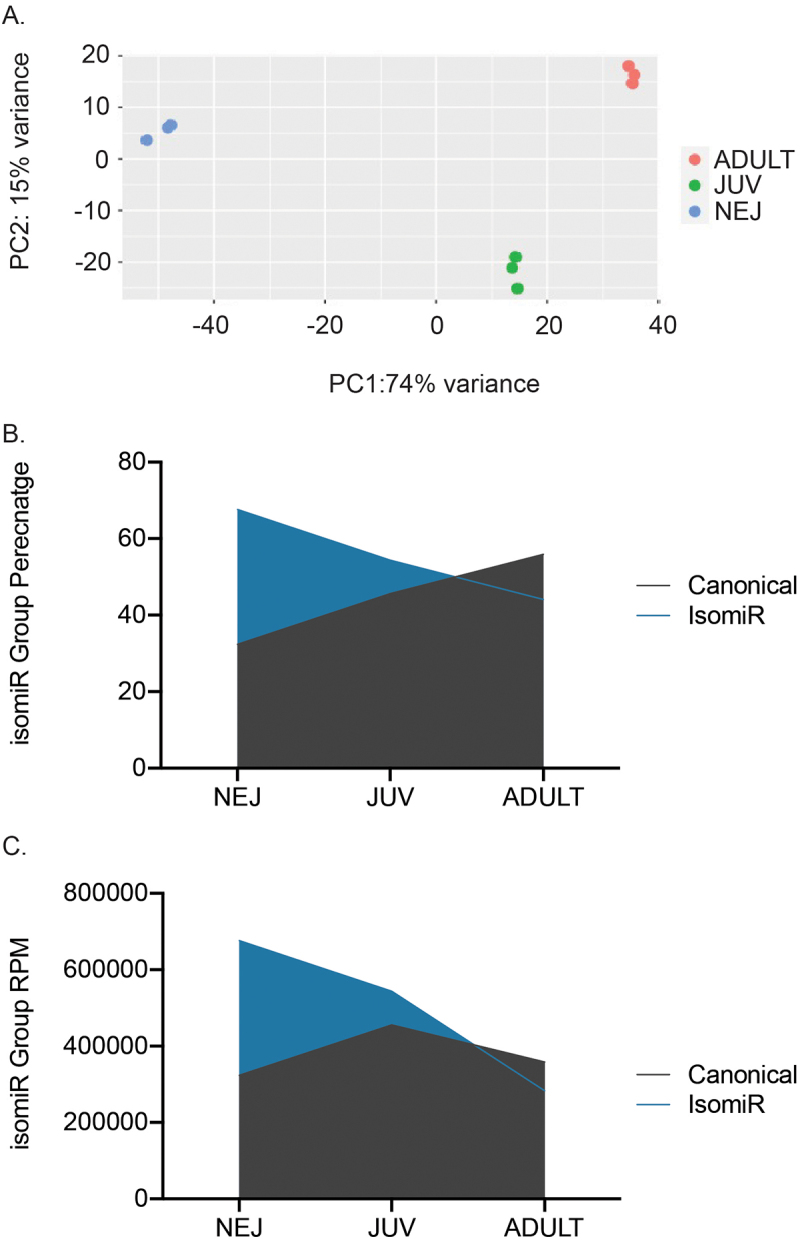
Fasciola hepatica miRNAs and isomiRs across developmental stages (A) Principal component analysis (PCA) plot of the isomiR expression across all life stages. Newly excysted juveniles (blue), 21-day juveniles (green), and adult parasites (red). Stacked area plots representing canonical miRNA and isomiR (B) relative abundance as a percentage of total rpm and (C) total reads (rpm) across the three life stages. Canonical reads (grey), and IsomiR reads (blue).

The three intra-mammalian life stages of *F. hepatica* development were subjected to miSeq analysis, and from this, isomiRs were found to be present in the newly excysted juveniles (NEJ 24hrs), immature 21-day juvenile flukes (JUV), and adult worms. For this study, we focused on isomiRs with end variations rather than internal polymorphisms such as SNPs. Using Principal Component Analysis (Figure 1A) (all canonical reads were removed from this analysis), the results showed a distinct separation of the three life stages when using isomiRs expression, with all biological triplicates for each life stage clustered together. This observation correlates with our previous findings that the canonical miRNA sequences of *Fasciola* show a defined temporal expression paralleling the parasites’ development [16].

Analysing the overall abundance of canonical vs isomiRs across each life stage (Figure 1B,C), revealed the presence of a greater number of isomiR reads (676,278 RPM) compared to canonical reads (323,379 RPM) at the NEJ life stage, which accounted for 68% of the total reads. The contribution of isomiRs to total read abundance decreases as the parasite matures to the adult stage with isomiRs accounting for 54% and 44% of total sequencing reads in JUV and adults, respectively. This represents a 24% decrease in isomiRs expression as the worms transition from NEJs to adult.

### 3`modified isomiRs are the dominant sequence variants

**Figure 2.**
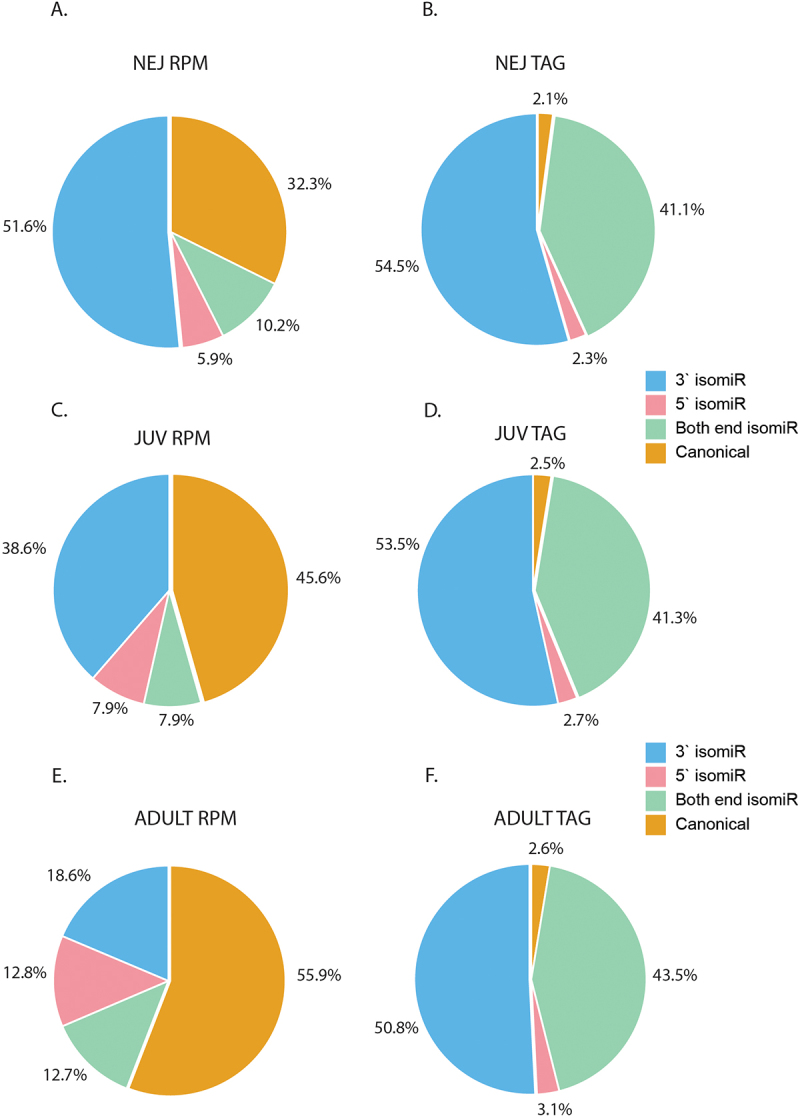
Distribution of templated canonical miRNAs and isomiR types across *F. hepatica* life stages: pie charts representing the relative abundance of canonical miRNAs and isomiRs as reads per million (rpm) for (A) NEJ, (C) JUV and (E) adults parasites and representing number of isomiRs or unique ‘tags’ for (B) NEJ, (D) JUV, and (F) adult flukes. IsomiRs were categorised based on end variation. Canonical miRNAs (orange), 3` end altered isomiRs (blue), 5` altered isomiRs (pink) and alterations to both ends (green).

The distribution of each isomiR type was then mapped across all three life stages, comparing read abundance and unique isomiR tags (Figure 2 and Supplementary file 1). In NEJs (Figure 2A,B), 3`end isomiRs are the predominant form in both overall abundance (51.6%) and unique tags (54.5%). Canonical miRNAs are the next most abundant (32.3%), followed by isomiRs with alterations to both ends (10.2%), and finally, the least abundant variant is the 5`end isomiRs (5.9%). As the NEJ develops to the adult stage, there is a significant decline in the abundance of 3` end isomiRs, with an opposing increase in the number of canonical miRNAs. Thus, in the juvenile (Figure 2C,D) and adult worms (Figure 2E,F), canonical miRNAs represent the most abundant form, accounting for 45.6% and 55.9%, respectively. For the isomiR variants, there was no change in the levels of either the 5`end and both 3’ and 5’ end isomiRs. These versions represented 7.9% of total miRNA sequences for juvenile worms and 12.7% for adults.

When analysing unique tags or the number of isomiRs, given the single sequence for each canonical miRNA, these represent a small percentage of all total miRNAs. Of the isomiRs, there was a higher number of unique isomiR sequences for 3`end isomiRs (~50%) followed by alterations to both ends (~40%) and 5`end isomiRs (~3%) consistently across all life stages (Figures 2B,D,F). Collectively, our findings suggest that isomiR expression is temporal and dependent on the worm life cycle. Importantly, isomiRs with alterations at the 5`end represented at least 10% of all miRNAs during worm development. This type of modification to the miRNA sequence can redirect regulation to different gene targets, thus potentially altering biological pathways.

**Figure 3.**
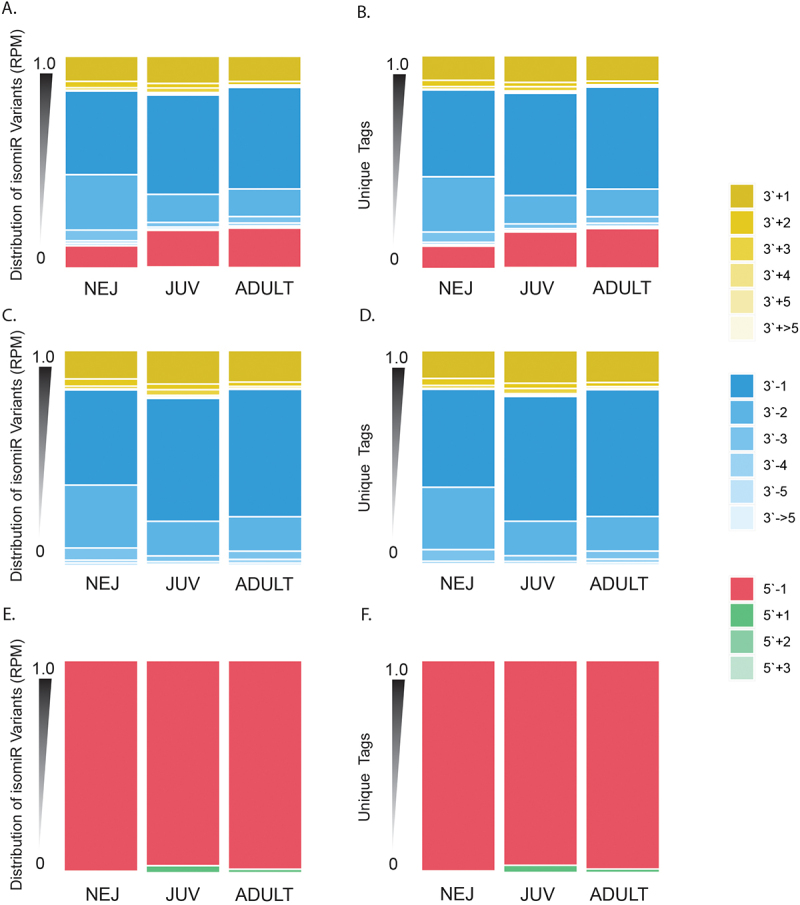
Annotation summary of isomiR end variation: characterisation of isomiR variation in terms of abundance (rpm) for (A) all isomiRs, (C) 3`end isomiRs and (E) 5` isomiRs. Summary of unique tags for (B) all IsomiRs, (D) 3` end isomiRs and (F) 5` end isomiRs. End nucleotide variation is categorised as 3` for alterations to the 3`end or 5` for alterations to the 5`end from canonical miRNAs. This is further categorised as elongation (+) or truncation (-); this is followed by a number that represents the number of nucleotides that are altered compared to the canonical structure.

We then characterised the nature of these modifications, whether they be extended (+) or truncated (-), and the number of nucleotide (nt) differences relative to the canonical sequence (Figure 3). This revealed that in the NEJ parasites, the dominant isomiR in terms of abundance (RPM) (Figure 3A) and unique tags (Figure 3B) was the 3`end truncation by a single nucleotide (~40%), followed by two nucleotides truncation (~26%). For both the JUV and adult worms, 3`end truncation by 1nt was the most abundant form (47% and 48%, respectively), followed by 5`end truncation by 1nt (17% and 19%) (Figure 3A). For the 5`end modifications, only extensions from 1–3 nt and truncations of 1nt were observed, with 5`end 1nt truncation isomiRs being the most common modification in terms of abundance (Figure 3E) and unique tags (Figure 3F) at all stages of development. In contrast, a broader range of 3`end modifications were observed with extensions/truncations ranging from 1nt to > 5 nt. While truncation by 1nt accounted for an average of 53% in all the 3’ end isomiRs (Figure 3C), there is a notable increase in the percentage of unique tags as the parasite develops (Figure 3D) from 45% in NEJ to 60% in JUV/Adult.

### Individual miRNA loci can produce isomiR variants across the different life stages

**Figure 4.**
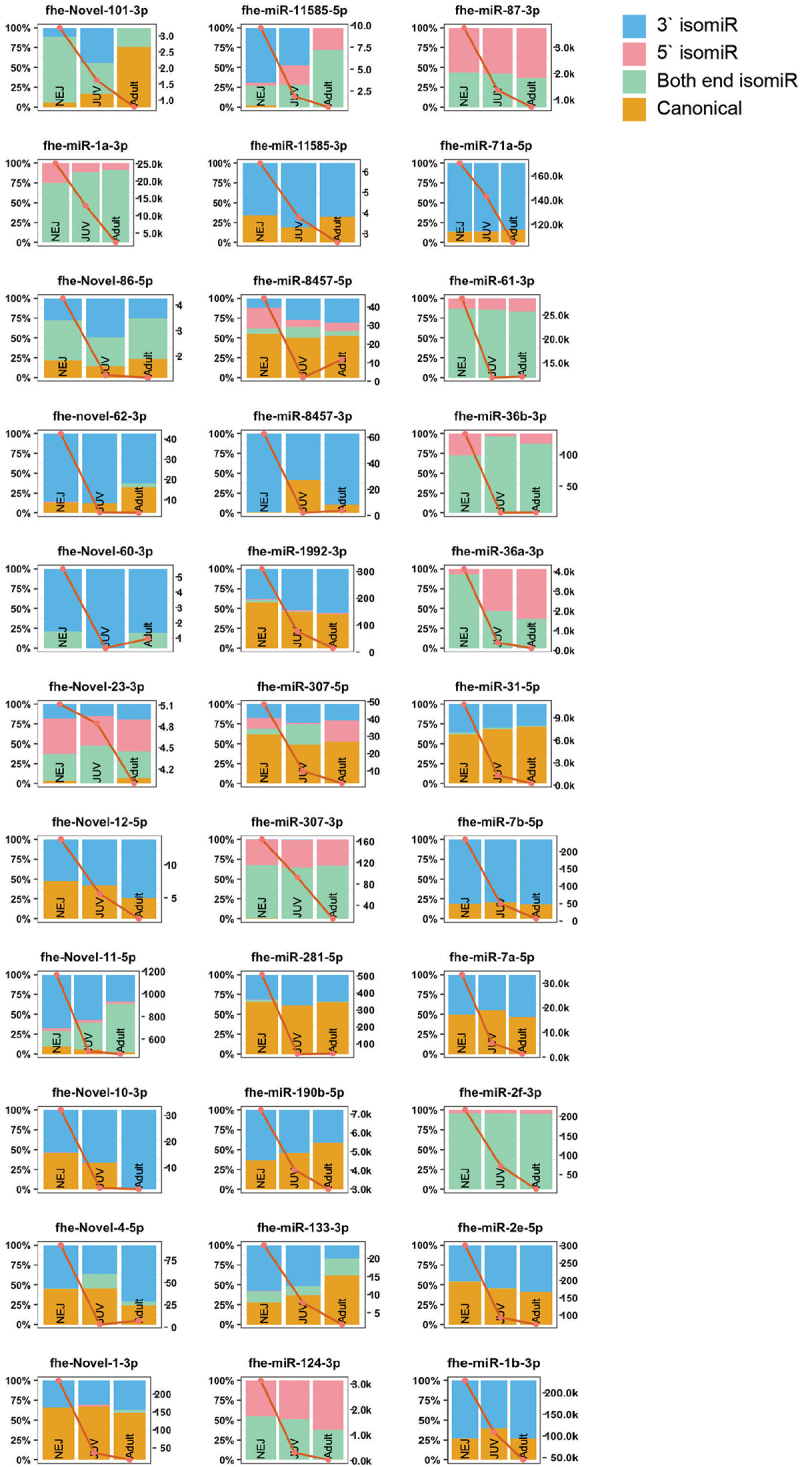
NEJ prominent miRNAs and their isomiR distribution and abundance (rpm): selected isomiRs with a greater relative read abundance (rpm) in the NEJ life stage compared to JUV and adults (right y-axis). Distribution of reads mapping to miRNA loci (left y-axis), with canonical reads (orange), isomiRs with 3`end alterations (blue), isomiRs with 5`end alterations (pink) and isomiRs with alterations at both ends (green). The red line represents the total abundance of miRNA loci mapped reads, including canonical miRNAs and isomiRs (rpm, right y-axis).

**Figure 5.**
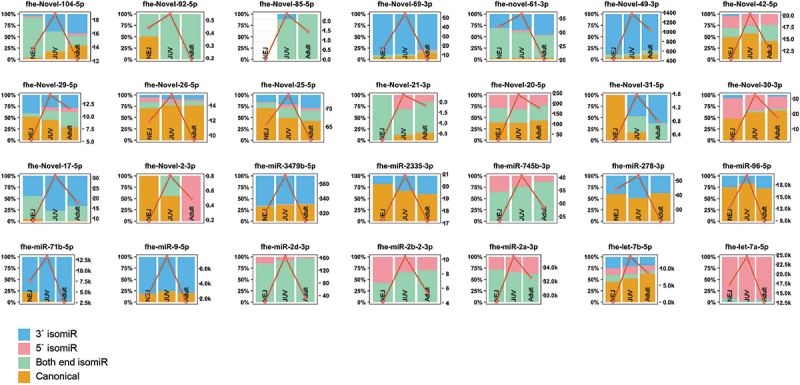
JUV isomiR distribution and abundance (rpm): selected isomiRs with a greater relative read abundance (rpm) in the JUV stage compared to NEJ and adults (right y-axis). Distribution of reads mapping to miRNA loci (left y-axis), with canonical reads (orange), isomiRs with 3`end alterations (blue), isomiRs with 5`end alterations (pink) and isomiRs with alterations at both ends (green). The red line represents the total abundance of miRNA loci mapped reads, including canonical miRNAs and isomiRs (rpm, right y-axis).

**Figure 6.**
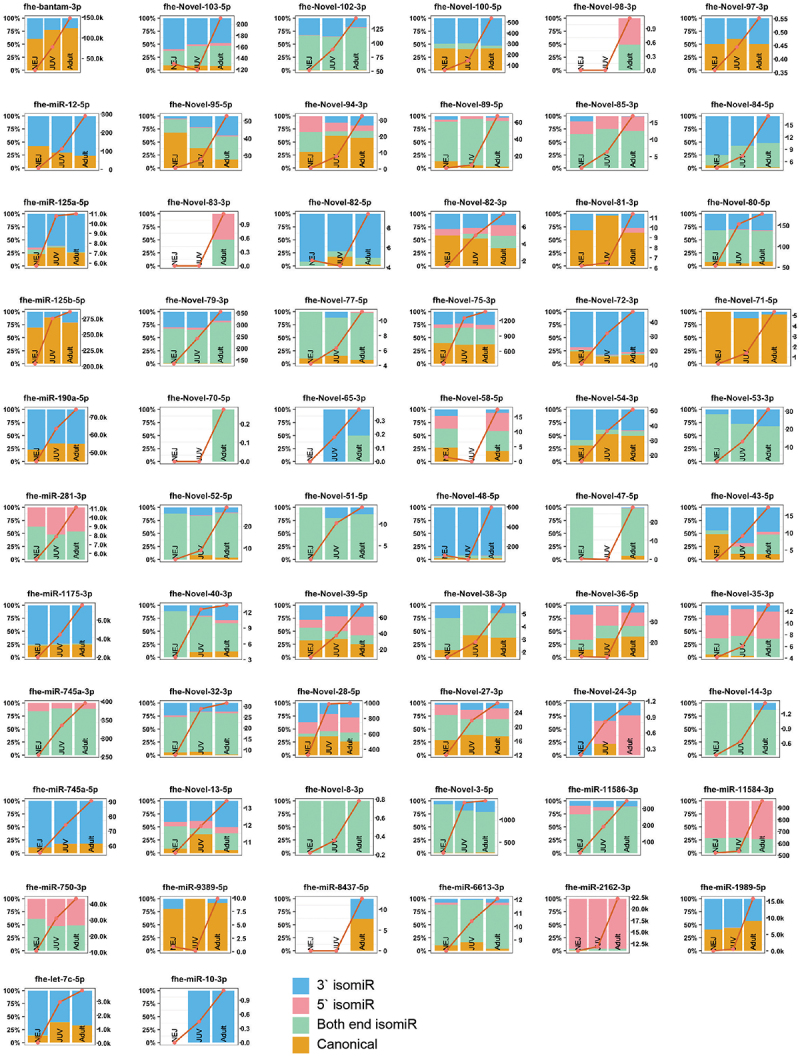
Adult miRNAs and their isomiR distribution and abundance (rpm) across life stages: selected isomiRs with a greater relative read abundance (rpm) in the adult life stage compared to NEJ and JUV (right y-axis). Distribution of reads mapping to miRNA loci (left y-axis), with canonical reads (orange), isomiRs with 3`end alterations (blue), isomiRs with 5`end alterations (pink) and isomiRs with alterations at both ends (green). The red line represents the total abundance of miRNA loci mapped reads, including canonical miRNAs and isomiRs (rpm, right y-axis).

We previously proposed that *Fasciola* miRNA expression is developmentally dependent and regulates different biological pathways specific to the requirements of each parasitic stage [16]. Thus, the possibility that stage-dependent miRNA loci could produce isomiR variants was investigated. To identify these specific miRNAs, we examined 191 miRNAs across the *Fasciola* developmental stages [25,26,29]. Of these, 159 were expressed in our cohort. From this dataset, miRNAs with the highest relative abundance in a single life stage were selected, resulting in 122 miRNAs showing temporal expression (Figures 4–6). The *Fasciola* miRNAs that did not display temporal expression using our cut-offs are depicted in Supplementary Figure S1.

In the NEJs, we identified 33 miRNAs that could be stage-specific (Figure 4). Among these, only three were predominantly expressed as canonical sequences, fhe-miR-31-5p, fhe-miR-281-5p, and fhe-novel-1-3p. The remaining miRNA sequences were represented by a higher abundance of isomiRs. Specifically, nine miRNAs showed an increased abundance ( > 50%) of 3′ end isomiRs (fhe-miR-1b-3p, fhe-miR-7b-5p, fhe-miR-71a-5p, fhe-miR-8457-3p, fhe-miR-11585-3p, fhe-novel-10-3p, fhe-novel-12-5p, fhe-novel-60-3p, and fhe-novel-62-3p), and two miRNAs exhibited a greater abundance of 5′ end isomiRs (fhe-miR-87-3p, fhe-miR-124-3p). Several miRNAs showed little to no expression of canonical sequences, including fhe-miR-1a-3p, fhe-miR-2f-3p, fhe-miR-36a-3p, fhe-miR-36b-3p, fhe-miR-61-3p, fhe-miR-87-3p, fhe-miR-124-3p, fhe-miR-307-3p, fhe-miR-11585-5p, and fhe-novel-11-5p.

The expression of some isomiRs exhibits distinct changes in their sequence preferences as the parasite matures. For example, for fhe-miR-133-3p, fhe-miR-190b-5p, and fhe-novel-101-3p, more isomiRs are observed in the NEJs, but as development progresses, there is a shift towards their canonical structures. Equally, fhe-miR-36a-3p, which shows a decrease in both-end isomiRs in NEJs, shifts to 5′ end isomiRs as the parasite matures.

For the JUV parasites, a total of 28 miRNAs were predominantly expressed (Figure 5). These miRNAs displayed high expression in JUVs compared to NEJs and adult worms. Five of these JUV miRNAs were present as canonical structures: fhe-miR-96-5p, fhe-miR-278-3p, fhe-miR-2335-3p, fhe-novel-30-3p, and fhe-novel-26-5p. In contrast, 19 miRNAs showed a greater expression of isomiR variants at the JUV stage. These included fhe-let-7a-5p, fhe-miR-2a-3p, fhe-miR-2b-2-3p, fhe-miR-2d-3p, fhe-miR-745-3p, fhe-miR-9-5p, fhe-miR-71b-5p, fhe-miR-3479b-5p, fhe-novel-21-3p, fhe-novel-17-5p, fhe-novel-20-5p, fhe-novel-48-3p, fhe-novel-42-5p, fhe-novel-61-3p, fhe-novel-29-5p, fhe-novel-104-5p, fhe-novel-92-5p, fhe-novel-85-5p, and fhe-novel-69-3p.

Of the adult parasite miRNAs (Figure 6), we identified 62 miRNAs that were predominantly expressed with this developmental stage with low expression in NEJ and JUVs but increased expression in adult worms. Of these, six were present as their canonical sequence: fhe-bantam-3p, fhe-miR-125b-5p, fhe-miR-9389-5p, fhe-novel-71-5p, fhe-novel-81-3p, and fhe-novel-97-3p. Whereas 50 miRNAs showed a higher abundance of isomiRs, and among these, 1 miRNAs exhibited little to no canonical structures. These included fhe-miR-10-3p, fhe-miR-281-3p, fhe-miR-745a-3p, fhe-miR-750-3p, fhe-miR-2162-3p, fhe-miR-11584-3p, fhe-miR-11586-3p, fhe-novel-83-3p, fhe-novel-65-3p, fhe-novel-70-5p, fhe-novel-53-3p, fhe-novel-51-5p, fhe-novel-79-3p, fhe-novel-85-3p, fhe-novel-102-3p, fhe-novel-98-3p, fhe-novel-14-3p, fhe-novel-8-3p, and fhe-novel-3-5p. The fhe-bantam-3p and fhe-miR-1989-5p exhibited an increase in the proportion of canonical structures over the developmental stages of the parasite, peaking in the adult worms.

### Worm isomiRs generated from Drosha and Dicer cleavage

The generation of isomiRs can occur by several different processes such as altered cleavage by Drosha or Dicer, or end alteration by terminal-nucleotide-transferase or 3`exonuclease activities [12,34–37]. To explore the variations in isomiR generation, we first classified miRNAs according to the site of any end alteration and the location of this change on the 3p or 5p arm (Supplementary Figure S2). To determine which isomiRs could be subjected to cleavage by either Drosha or Dicer, we categorised each isomiR sequence based on its location and alterations on the precursor arms (Supplementary Figure S2A).

IsomiRs located on the 3p arm were classified as 3`Drosha if they had alterations at the 3`end, and as 5`Dicer if they had alterations at the 5`end. Similarly, isomiRs located on the 5p arm were categorised as 5`Drosha if they had 5`end alterations, and as 3`Dicer if they had 3`end alterations. Using this filter, we found no difference in the number of alternative cleavage events produced by Drosha (on average 44.6% across stages) or Dicer (average 55.4% across stages) (Supplementary Figure S2B). However, examining isomiR abundance (Supplementary Figure S2C), suggests that Dicer generates more cleavage events (65% on average across life stages) compared to Drosha (35% on average across life stages).

**Figure 7.**
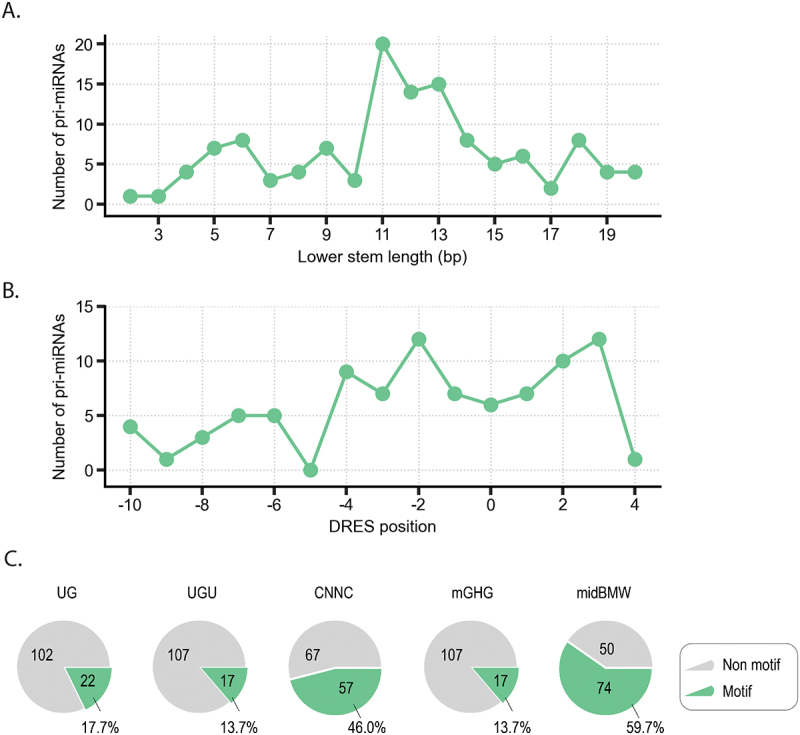
Identification of Drosha binding motifs in *F. hepatica* pri-miRNA sequences: A) lower stem length of pri-miRNAs. B) position of Drosha dsRNA recognition sites in pri-miRNA. C) abundance of binding motifs in pri-miRNAs.

To further elucidate the cleavage activity of Drosha, we investigated the abundance of Microprocessor motifs in *F. hepatica* primary miRNAs. First, 180 pri-miRNA sequences were extracted from the *Fasciola* genome PRJEB25283, by extending 20 nt on either side of the reported pre-miRNA sequences [25,26,29]. The structure of each pri-miRNA was predicted using RNAfold tool [32] (Supplementary file 2). We selected 124 pri-miRNAs without multiple loops for further analysis. Then, for each pri-miRNAs we examined the lower stem and Microprocessor motifs including UG, UGU, mGHG, CNNC, midBMW, and DRES (Figure 7).

*F. hepatica* presents a unique miRNA profile with enrichment in lower stem of 11–13 bp with 11 bp appearing as the most abundant (Figure 7A). This contrasts with humans (primarily 13-bp lower stems) and *C. elegans* (primarily shorter lower stems) [38]. The Drosha dsRNA recognition sites (DRES), typically used and recognised by Drosha during non-canonical processing of short lower stem pri-miRNAs, were not enriched within the *F. hepatica* sequences. Instead, Microprocessor motifs such as CNNC, UG, UGU, mGHG and midBMW were most abundant in the parasite pri-miRNAs, which parallels to the motif distribution in humans (Figure 7B,C) [11,33,38–40]. This profile suggests that *F. hepatica* pri-miRNAs share a closer resemblance to human miRNAs than those in the free-living parasite *C. elegans*. This intriguing observation prompts speculation about the potential mechanisms to enhance the infection capacity of *F. hepatica* in mammals, as well as the mechanisms guiding the precise cleavage events that lead to the generation of isomiRs.

### Untemplated isomiRs generated from terminal-nucleotide-transferase

**Figure 8.**
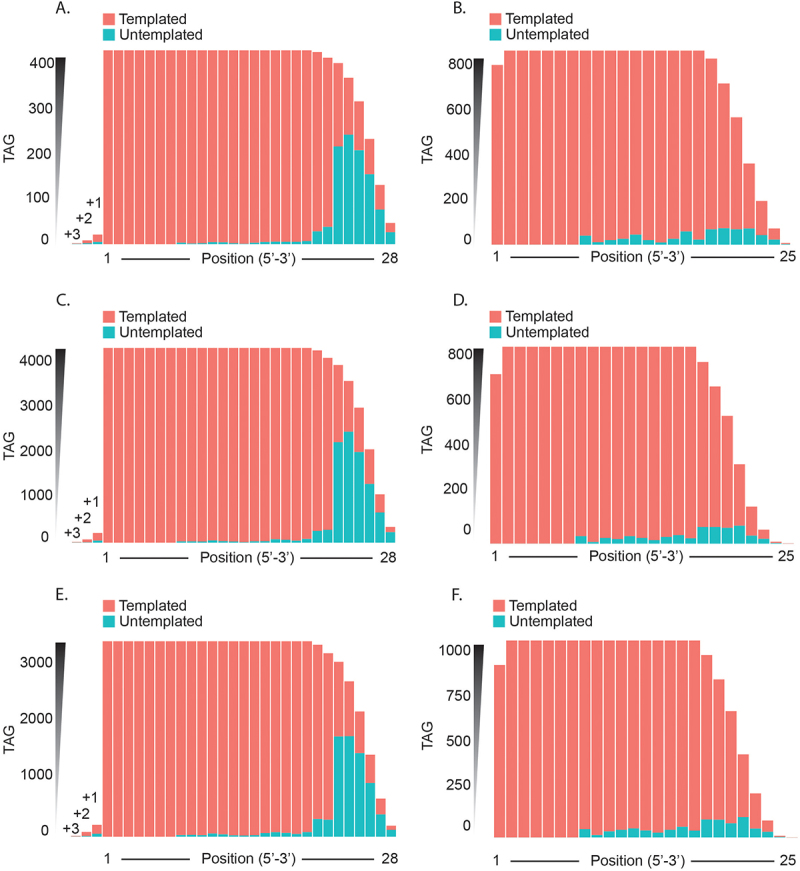
Templated vs untemplated isomiRs: characterisation of each isomiR nucleotide as templated or untemplated compared to the pri-miRNA for (A, C, E) extended isomiRs and (B, D, F) truncated isomiRs. Represented as a summary of unique tags for (A, B) NEJs, (C, D), JUVs and (E, F) adult parasites.

Posttranscriptional processing by terminal nucleotide transferases on isomiRs often results in the addition of nucleotides at either end. In this study, we have denoted nucleotide additions and truncations with a (+) and (−) sign, respectively. Our analysis (Figure 8) showed that there were within the *F. hepatica* isomiR sequences fewer nucleotide additions to the 5′ end (up to 3 nucleotides) compared to the 3′ end (up to 8 nucleotides).

For the 5′ end-extended isomiRs, the majority of nucleotides at each position are templated in NEJs (Figure 8A), JUVs (Figure 8C), and adults (Figure 8E). Specifically, in NEJs, templated nucleotides accounted for 77%, 77%, and 61% at positions 5′+1, 5′+2, and 5′+3 extensions, respectively; in JUVs, these were 79%, 92%, and 67%; and in adults, 76%, 89%, and 69%. However, when investigating the 3′ end-extended isomiRs, we consistently observed a majority of untemplated nucleotide additions across all three life stages. As nucleotides are added, the proportion of templated nucleotides decreases with length. Beyond the 22nd position, the percentage of templated nucleotides decreases from approximately 46% to 30%. It should be noted that this data represents *F. hepatica* miRNAs, which vary in length from 20 to 25 nucleotides.

Our analysis also included end variant isomiRs with internal nucleotide variations (excluding the seed region). For isomiRs with nucleotide extensions, internal nucleotide variations (from positions 8 to 21) were minimal in NEJs (Figure 8A) and JUVs (Figure 8C), ranging from 0.2% to 7% untemplated nucleotides. In adult parasites (Figure 8E), we observed a slight increase, with untemplated nucleotides ranging from 0.6% to 9%. The isomiRs with truncations at either end showed a greater percentage of untemplated internal nucleotide changes, ranging from 0.8% to 12% across the three life stages in NEJs (Figure 8B), JUVs (Figure 8D), and adults (Figure 8F).

**Figure 9.**
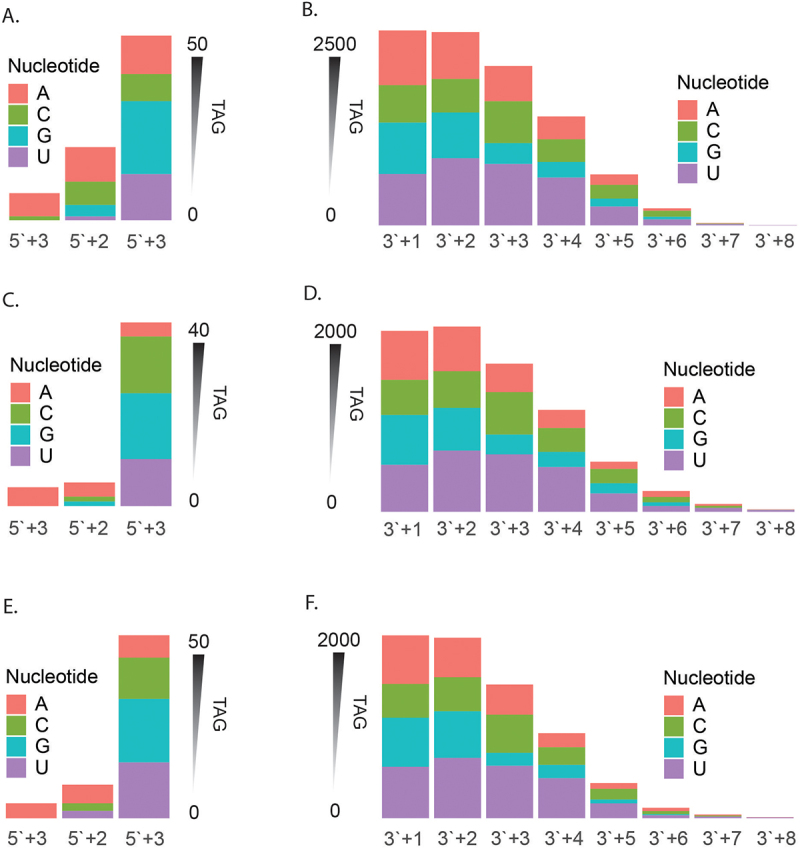
Nucleotide additions for extended isomiRs: characterisation of nucleotide additions for all extended isomiRs at the (A, C, E) 5`end and (B, D, F) 3`end. Represented as a summary of unique tags for (A, B) NEJs, (C, D), JUVs and (E, F) adult parasites.

We then characterizsd the nucleotide additions for untemplated extended isomiRs at each position, comparing the 5′ and 3′ end extensions across NEJs, JUVs, and adult stages (Figure 9A–F). At the 5′ end (Figures 9A,C,E), adenosine (A) was predominantly added at the 3nt extension position, accounting for 86% in NEJs and 100% in both JUVs and adults. This trend was less pronounced at the 2nt extension, where adenosine represented 47%, 60%, and 55% in NEJs, JUVs, and adults, respectively. For the 1nt extension, guanine (G) was more frequent, making up 40%, 36%, and 35% of the extended nucleotides in NEJs, JUVs, and adults.

In contrast, at the 3′ end (Figure 9B,D,F), nucleotide distribution was similar for the 1nt extension, with each nucleotide contributing approximately 25% across all stages. However, as the extension length increased, uracil (U) became the dominant nucleotide. In NEJs, uracil accounted for 26% at the 1nt extension and increased to 67% at the 8nt extension. Similar patterns were observed in JUVs and adults, with uracil’s contribution increasing from 26% at 1nt to 80% at 8nt in adults. Guanine (G) and cytosine (C) contributed similarly to adenosine across most extension positions, suggesting that these nucleotides play a significant role in isomiR modifications across the parasite’s development. In the next analysis, we then investigated changes at the 5`end.

### 5` end alterations can redirect mRNA targets to alter regulation of biological pathways

At the 5`end, any alterations will change the seed sequence, thereby redirecting these isomiRs to different gene targets. For the 167 detected miRNAs, 147 had at least one IsomiR with a 5`end alterations resulting in seed shifting (123 classified as 5`IsomiR and 141 as both end IsomiR). Filtering all *F. hepatica* isomiRs with 5′ end alterations that had expression levels above 5000 RPM, resulted in the identification of six isomiRs, all with a single nucleotide truncation at the 5′ end. Further refinement, based on the presence of canonical sequences in the sequencing data across life stages (to eliminate potential mis-annotations), narrowed this list to three 5′ truncated isomiRs, fhe-miR-2162-3p, fhe-miR-1a-3p and fhe-miR-281-3p. For all three isomiRs, the truncated variants were more abundant than their corresponding canonical miRNA sequences, suggesting that the isomiR could be the dominant form.

**Figure 10.**
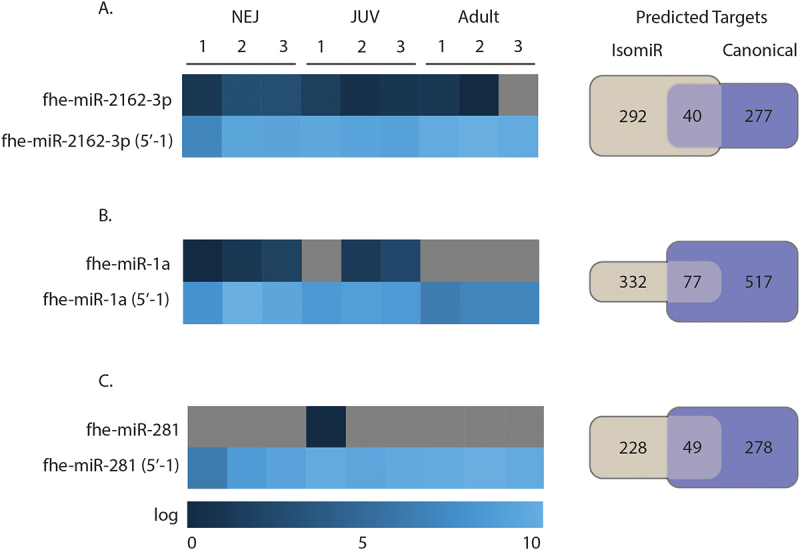
Target prediction of canonical and 5`end altered isomiRs. Expression of selected 5`end altered isomiRs in three biological samples and the number of predicted targets for the canonical miRNAs and their 5`end altered isomiRs for A) fhe-miR-2162-3p, B) fhe-miR-1a-3p and C) fhe-miR-281-3p. On the left panel a heat map showing the expression of these miRNAs. On the right panel is a Venn diagram showing which targets are in common and unique for each conical and isomiR.

To evaluate their functionality, we performed target prediction analyses for both the canonical miRNA sequences and their 5′ isomiRs using TargetScan and miRanda (Figure 10, Supplementary File 3). As expected, the single nucleotide truncation at the 5′ end shifted the seed sequence, resulting in distinct gene target profiles (Figure 10). fhe-miR-1a-3p showed the largest difference between its canonical form (517 unique targets) and its isomiR (332 unique targets), with only 77 shared targets. For fhe-miR-2162-3p, 40 genes were predicted to be common between the canonical and isomiR forms, while the canonical sequence had 277 unique predicted targets and the isomiR had 292. The canonical form of fhe-miR-281-3p was detected only in the juvenile stage, with 278 unique targets, while its isomiR could target 228 unique genes. There were 49 common gene targets predicted for both forms.

**Figure 11.**
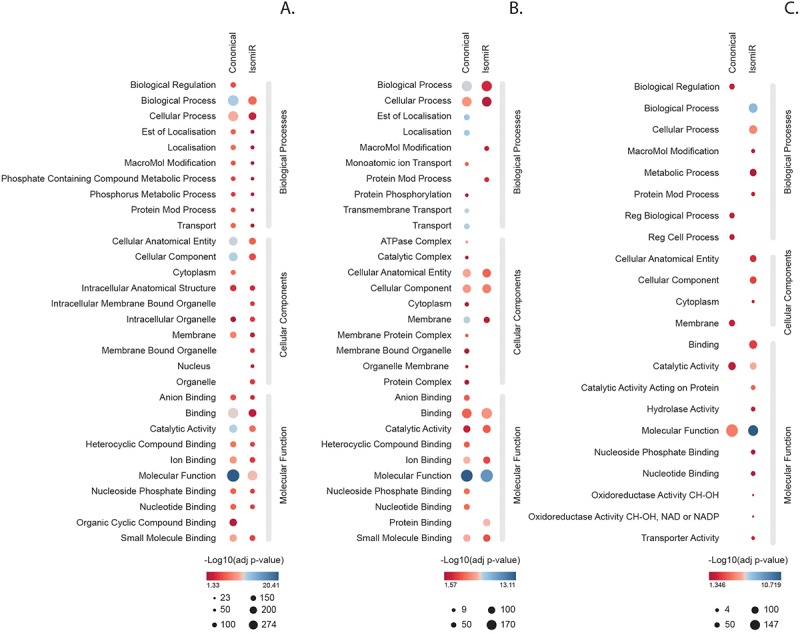
Target prediction of canonical and 5`end altered isomiRs. Predicted targets for the canonical miRNAs and their 5`end isomiRs for A) fhe-miR-1a-3p, B) fhe-miR-2162-3p and C) fhe-miR-281-3p.

We next evaluated whether the 5′ isomiRs differentially regulated biological pathways, as compared to the canonical sequences, by redirecting gene targets. To do this, all *F. hepatica* miRNA target genes were annotated using Gene Ontology (GO) terms derived from corresponding gene sequences in the PREJEB25283 and PRJEB6687 genomes (Supplementary File 4). We then compared the GO terms between the canonical miRNAs and their isomiR variants. For fhe-miR-1a-3p (Figure 11A), no differences were observed in GO pathways between the canonical form and the isomiR, despite it having the highest number of unique predicted targets. In contrast, fhe-miR-2162-3p (Figure 11B) showed clear evidence of target redirection by the isomiR variant, resulting in the loss of 17 pathways, especially within cellular components and biological processes. Similarly, target redirection was observed for fhe-miR-281-3p, where the 5′ isomiR led to a distinct gain of 15 GO pathways, particularly in molecular function (Figure 11C). For this analysis, we applied a stringent cut-off based on RPM and expression across all stages. Without this cut-off, 123 out of 159 expressed miRNAs would have shown at least one 5′ isomiR variant.

## Discussion

In this study, we categorised the isomiR profile of the parasitic worm *Fasciola hepatica* using our miRNA sequencing data sets of three intra-mammalian developmental life stages; newly excysted juveniles (NEJ), Juveniles (JUV), and adults. We found a distinct shift in isomiR distribution across the maturation of the parasite, with a greater number and abundance of isomiR in the NEJs. In many cases, for individual miRNA sequences, the isomiRs were the most dominant form, however as the parasite develops into the adult life stage, there is a switch to the canonical forms of miRNA sequences. Based on this analysis, we can propose that isomiR expression is temporal and dependent on the parasitic life cycle.

### Characterisation of temporal isomiRs in development

There are very few studies investigating the developmental expression of isomiRs. In *Drosophila melanogaster* [41] and zebrafish [42], isomiRs were found to be differentially expressed across development. For several *Drosophila* isomiRs, their expression was tied to a specific life cycle or/and to a tissue-dependent manner [41]. In these flies, non-templated nucleotide additions of Adenosine to the 3`end were highly abundant in early development and showed enrichment for target sites in developmental genes that are expressed during late embryogenesis. Similarly, in zebrafish, changes in isomiR expression were observed throughout gonadal development, with nucleotide additions being the most frequent [25]. In contrast, for *F. hepatica*, truncated isomiRs were more abundant. Additionally, Uracil was the most overrepresented nucleotide addition at the 3`end, and Adenosine was most prevalent at the 5`end. It is possible that non-templated nucleotide additions may increase miRNA stability or strengthen miRNA target interactions [41].

*Fasciola* is one of the few parasitic worms that can infect a wide range of mammalian hosts. The increased abundance and diversity of isomiRs during the NEJ stage may suggest that *Fasciola* requires a broad range of miRNA variants to provide regulatory flexibility [4], allowing it to respond to the specific conditions of its host. As the parasite matures, we see a return to canonical miRNAs, suggesting that development drives the production of isomiRs and canonical versions. In contrast for the non-parasitic free-living worm *C. elegans*, it was shown that most miRNA loci did not produce a substantial fraction of templated isomiRs, with canonical miRNA sequences being the dominant form [30]. This expression is in stark difference to the profile for *F. hepatica* and further supports the idea that the increase in abundance of isomiRs, during the early infective stages, supports the parasitic lifestyle and adaptation to life inside a mammalian host.

IsomiRs have been reported to be loaded onto Ago, demonstrating a functional role and ability to bind targets [43]. Target comparison studies have revealed that isomiRs can work cooperatively with their canonical counterparts [4], have a cooperative effect through different pathways [44] and have divergent functions [45]. IsomiRs at the 3`end are the most abundant form in *F. hepatica*, consistent with other studies both animals and plants [10,12,46,47].

### The biogenesis of *Fasciola* IsomiRs

Analysis of *F. hepatica* genome and proteome sequences has revealed the parasite possesses microRNA processing enzymes, including Drosha and Dicer, like other parasitic flatworms [48]. We explored the idea that imprecise cleavage by Drosha and Dicer was producing isomiRs with altered lengths [10]. Drosha and Dicer have defined cleavage sites; however, the structural characteristics [49] and alterations to pri-miRNA sequences can compromise the catalysis efficiency and cleavage [50,51]. Although there was no difference between the number of Drosha and Dicer-predicted cleavage events across the development of *F. hepatica*, there was a higher abundance (RPM) of isomiRs with Dicer cleavage events compared to Drosha. This suggests that Dicer-mediated cleavage may play a more prominent role in generating isomiRs for this parasite.

We also noted that the Drosha dsRNA recognition sites (DRES) found in *F. hepatica* pri-mRNAs are more like those seen in mammalian pri-miRNAs, rather than *C. elegans*. This suggests that *F. hepatica* has developed a mammalian-like mode of miRNA processing, which indicates an adaptation for communication with the host. Indeed, we have previously reported that *Fasciola* miRNAs are loaded on Ago2 with their host macrophages to become functionalised miRNAs [52].

Post-transcriptional modifications to the canonical miRNA can also generate isomiR variants [10]. Crystallographic studies of Argonaute proteins showed that 5`ends of miRNAs are buried within the MID domain, while 3`ends extend from the PAZ domain and are exposed to exonucleolytic attack, which may cause miRNA shortening [53,54]. For example, 3`-5` exoribonucleases have been shown to shorten the 3`end of Ago-bound miRNAs in Drosophila [36]. *Fasciola hepatica* possesses gene sequences that align with the exonucleases ERI1, ERI2 and ERI3 from the closely related parasitic flatworm *Schistosoma mansoni* [48], suggesting that they may be involved in the generation of these isomiRs.

Non-templated isomiRs, where the nucleotide sequences differ from the canonical miRNA [10], may arise due to posttranscriptional enzymatic modifications, such as the addition of nucleotides by nucleotidyl transferases, including uridyltransferases and adenyltransferases [12]. Additionally, polymorphic isomiRs, which contain altered internal nucleotide sequences, could be the result of A-to-I editing by ADAR enzymes [10]. There are no equivalent genes within the *Fasciola hepatica* genome, and it remains an unknown as to how non-templated isomiRs are being generated.

### Target redirection by *Fasciola* 5`end isomiRs

For *Fasciola*, isomiRs with alterations at the 5`end represented up to 10% of RPM of all miRNAs during development. Modification at the 5`end can redirect miRNAs to different targets and may lead to the regulation of alternative pathways. This was evident for the three highly expressed 5’ isomiR variants: fhe-miR-1a-3p, fhe-miR-2162-3p, and fhe-miR-281-3p. Each of these isomiRs has a single nucleotide truncation at the 5’ end and displayed some degree of stage-specific expression.

Many of the predicted targets for these isomiRs are linked to metabolic activity, which is of interest as this is a critical element of the parasite’s development. During the NEJ phase, the parasite transitions from glycogen catabolism to glucose synthesis and glycolysis, followed by a switch to anaerobic metabolism in the JUV and adult stages as oxygen becomes limited [22,24,27]. The regulation of key metabolic pathways such as glycolysis, gluconeogenesis, the tricarboxylic acid (TCA)/Krebs cycle, and oxidative phosphorylation appears to be closely linked to the observed isomiR expression.

For example, fhe-miR-1a-3p isomiR regulates genes involved in aerobic metabolism (e.g. NADH-ubiquinone oxidoreductase) and the TCA cycle (e.g. isocitrate dehydrogenase), as well as enzymes in glycolysis and the pentose phosphate pathway, such as 6-phosphogluconate dehydrogenase and glutathione dehydrogenase. Similarly, fhe-miR-281-3p isomiR targets genes related to anaerobic metabolism, such as lactate dehydrogenase, while both isomiRs regulate isocitrate dehydrogenase (NAD+), a key player in the TCA cycle. This ability of isomiRs to regulate genes in both aerobic and anaerobic metabolic pathways highlights a role in fine-tuning gene expression during worm development.

In addition to their metabolic targets, these three isomiRs are also predicted to regulate developmental genes. For example, fhe-miR-1a-3p targets FhLeg4, which is not expressed during the NEJ stage but becomes active as the parasite matures [24]. This expression pattern may be influenced by the increased abundance of fhe-miR-1a-3p isomiRs in NEJs. Although the specific function of FhLeg4 remains elusive, its family members, FhLeg1, FhLeg2, and FhLeg5, are known to be activated during late parasite development and are associated with mature blood feeding.

Similarly, the fhe-miR-281-3p isomiR is predicted to target the juvenile-specific gene FhCL2, which is crucial for tissue degradation, migration, and feeding in mammalian hosts [27]. Furthermore, the Kunitz-type protease inhibitor targeted by the fhe-miR-2162-3p isomiR is regulated throughout the life stages, with peak expression in NEJs [55]. This inhibitor family is believed to regulate parasite cathepsin L proteases and impair host immune cell activation, aiding in parasite survival.

Beyond these developmental genes, several other pathways essential for parasitic survival were predicted to be regulated. The regulation of eukaryotic initiation factor-4E by fhe-miR-281-3p suggests an influence on stress-response mechanisms, potentially enhancing the parasite’s survival during infection [16]. This protein is known to bolster stress resilience, particularly critical in the NEJ stage, where overcoming environmental stressors is vital for survival and successful progression to later stages.

In addition to altering the gene target specificity, changes to the type of 5` nucleotide have been shown to direct miRNAs to specific Argonauts [56,57]. In plants, miRNAs with a 5’ uracil (5’U) are directed to Ago1, 5’ adenine (5’A) to Ago2, and 5’ cytosine (5’C) to Ago4 and Ago5 [58,59]. As *F. hepatica* has been predicted to possess three Ago proteins [48], it is possible that one purpose of the 5’ isomiRs is also to guide miRNAs to different Ago proteins for other functional roles.

## Conclusion

The diversification of miRNA sequences through the production of isomiRs in parasitic worms is likely an adaptation for survival and reproduction within their mammalian hosts. Unlike the free-living worm *C. elegans*, which predominantly produces canonical miRNAs, the parasitic worm *F. hepatica* has evolved a more diverse miRNA regulatory system. This isomiR diversity may enable the parasite to dynamically regulate gene expression in response to the complex and variable conditions encountered within mammalian hosts. These isomiRs also broaden the target repertoire, enabling the parasite to manipulate a wide array of host cellular pathways. The production of isomiRs would increase regulatory plasticity, providing the parasite with the ability to fine-tune its gene expression during different stages of infection or when infecting various mammalian species. To confirm this possibility, a direct comparison between free-living and parasitic worms and a comparison between different species of parasitic worms is required. This will confirm whether the observed shifts in isomiR expression are unique to *F. hepatica* or part of a common adaptation by parasitic worms.

Our data suggests that the generation of isomiRs in *Fasciola* may represent a key evolutionary advantage for parasitic worms. These isomiRs may serve as developmental switches that permit the successful progression of the infection cycle and sustain long-term infection. However, direct evidence through inhibitory/knockdown or overexpression studies will be required to validate a functional role. In addition, an exploration into the signals, environmental changes, and host cells that might influence the change in isomiR expression is required to fully elucidate the intersection between biological and molecular pathways that underpin parasite growth and maturation. This type of experimental analysis is only possible now with the recent emergence of organoid and spheroid 3D culture systems [60,61] that support the *in* *vitro* growth of helminths and replace the requirement for mammalian hosts. In light of the expanding body of miRNA data, this study clearly demonstrates that isomiRs play a significantly more critical role in parasitism than perhaps previously recognized.

## Supplementary Material

Data File 1 QC Report.pdf

Supplementary file 3.xlsx

Supp Fig 1.jpg

Suppl Fig 2.pdf

Supplementary file 2.xlsx

Supplementary file 4.xlsx

Supplementary file 1.xlsx

## Data Availability

All miR-Seq Fastq files are freely available and deposited in NCBI’s Gene Expression Omnibus; accession number GSE186948. All analysed data generated during this study is included in this published article [and its supplementary information files].

## References

[1] Bartel DP. Metazoan micrornas. Cell. 2018;173(1):20–51. doi: 10.1016/j.cell.2018.03.006 29570994 PMC6091663

[2] Lee RC, Feinbaum RL, Ambros V. The C. elegans heterochronic gene lin-4 encodes small RNAs with antisense complementarity to lin-14. Cell. 1993;75(5):843–854. doi: 10.1016/0092-8674(93)90529-y 8252621

[3] Chen X, Rechavi O. Plant and animal small RNA communications between cells and organisms. Nat Rev Mol Cell Biol. 2022;23(3):185–203.doi: 10.1038/s41580-021-00425-y 34707241 PMC9208737

[4] Cloonan N, Wani S, Xu Q, et al. MicroRNAs and their isomiRs function cooperatively to target common biological pathways. Genome Biol. 2011;12(12):R126. doi: 10.1186/gb-2011-12-12-r126 22208850 PMC3334621

[5] Ha M, Kim VN. Regulation of microRNA biogenesis. Nat Rev Mol Cell Biol. 2014;15(8):509–524. doi: 10.1038/nrm3838 25027649

[6] Shang R, Lee S, Senavirathne G, et al. microRNAs in action: biogenesis, function and regulation. Nat Rev Genet. 2023;24(12):816–833. doi: 10.1038/s41576-023-00611-y 37380761 PMC11087887

[7] Landgraf P, Rusu M, Sheridan R, et al. A mammalian microRNA expression atlas based on small RNA library sequencing. Cell. 2007;129(7):1401–1414. doi: 10.1016/j.cell.2007.04.040 17604727 PMC2681231

[8] Tomasello L, Distefano R, Nigita G, et al. The MicroRNA family gets wider: the IsomiRs classification and role. Front Cell Dev Biol. 2021;9:668648. doi: 10.3389/fcell.2021.668648 34178993 PMC8220208

[9] Gong J, Tong Y, Zhang HM, et al. Genome‐wide identification of SNPs in microRNA genes and the SNP effects on microRNA target binding and biogenesis. Hum Mutat. 2012;33(1):254–263. doi: 10.1002/humu.21641 22045659

[10] Neilsen CT, Goodall GJ, Bracken CP. IsomiRs–the overlooked repertoire in the dynamic microRnaome. Trends In Genet. 2012;28(11):544–549. doi: 10.1016/j.tig.2012.07.005 22883467

[11] Li S, Nguyen TD, Nguyen TL, et al. Mismatched and wobble base pairs govern primary microRNA processing by human microprocessor. Nat Commun. 2020;11(1):1926. doi: 10.1038/s41467-020-15674-2 32317642 PMC7174388

[12] Wyman SK, Knouf EC, Parkin RK, et al. Post-transcriptional generation of miRNA variants by multiple nucleotidyl transferases contributes to miRNA transcriptome complexity. Genome Res. 2011;21(9):1450–1461. doi: 10.1101/gr.118059.110 21813625 PMC3166830

[13] Manzano M, Forte E, Raja AN, et al. Divergent target recognition by coexpressed 5′-isomiRs of miR-142-3p and selective viral mimicry. RNA. 2015;21(9):1606–1620. doi: 10.1261/rna.048876.114 26137849 PMC4536321

[14] Olejniczak M, Kotowska-Zimmer A, Krzyzosiak W. Stress-induced changes in miRNA biogenesis and functioning. Cell Mol Life Sci. 2018;75(2):177–191. doi: 10.1007/s00018-017-2591-0 28717872 PMC5756259

[15] Wagner V, Meese E, Keller A. The intricacies of isomiRs: from classification to clinical relevance. Trends Genet. 2024;40(9):784–796. doi: 10.1016/j.tig.2024.05.007 38862304

[16] Ricafrente A, Cwiklinski K, Nguyen H, et al. Stage-specific miRnas regulate gene expression associated with growth, development and parasite-host interaction during the intra-mammalian migration of the zoonotic helminth parasite fasciola hepatica. BMC Genomics. 2022;23(1):419. doi: 10.1186/s12864-022-08644-z 35659245 PMC9167548

[17] Barrero-Torres DM, Herrera-Torres G, Perez J, et al. Unraveling the microRNAs involved in fasciolosis: master regulators of the host–parasite crosstalk. IJMS. 2024;26(1):26. doi: 10.3390/ijms26010204 39796061 PMC11719827

[18] Ojo OE, Kreuzer-Redmer S. MicroRNAs in ruminants and their potential role in nutrition and physiology. Vet Sci. 2023;10(1):57. doi: 10.3390/vetsci10010057 36669058 PMC9867202

[19] Zhou X, Hong Y, Shang Z, et al. The potential role of MicroRNA-124-3p in growth, development, and reproduction of schistosoma japonicum. Front Cell Infect Microbiol. 2022;12:862496. doi: 10.3389/fcimb.2022.862496 35493736 PMC9043613

[20] Sun C, Luo F, You Y, et al. MicroRNA-1 targets ribosomal protein genes to regulate the growth, development and reproduction of schistosoma japonicum. Int J Parasitol. 2023;53(11–12):637–649. doi: 10.1016/j.ijpara.2023.03.007 37355197

[21] Webb CM, Cabada MM. Recent developments in the epidemiology, diagnosis, and treatment of fasciola infection. Curr Opin Infect Dis. 2018;31(5):409–414. doi: 10.1097/QCO.0000000000000482 30113327

[22] Cwiklinski K, Jewhurst H, McVeigh P, et al. Infection by the helminth parasite fasciola hepatica requires rapid regulation of metabolic, virulence, and invasive factors to adjust to its mammalian host. Mol & Cellular Proteom. 2018;17(4):792–809. doi: 10.1074/mcp.RA117.000445 PMC5880117 29321187

[23] McCusker P, McVeigh P, Rathinasamy V, et al. Stimulating neoblast-like cell proliferation in juvenile fasciola hepatica supports growth and progression towards the adult phenotype in vitro. PLOS Negl Trop Dis. 2016;10(9):e0004994. doi: 10.1371/journal.pntd.0004994 27622752 PMC5021332

[24] Cwiklinski K, Dalton JP, Dufresne PJ, et al. The fasciola hepatica genome: gene duplication and polymorphism reveals adaptation to the host environment and the capacity for rapid evolution. Genome Biol. 2015;16(1):71. doi: 10.1186/s13059-015-0632-2 25887684 PMC4404566

[25] Fontenla S, Langleib M, de la torre-Escudero E, et al. Role of fasciola hepatica small RNAs in the interaction with the mammalian host. Front Cell Infect Microbiol. 2021;11:812141. doi: 10.3389/fcimb.2021.812141 35155272 PMC8824774

[26] Herron CM, O’Connor A, Robb E, et al. Developmental regulation and functional prediction of microRNAs in an expanded fasciola hepatica miRnome. Front Cell Infect Microbiol. 2022;12:811123. doi: 10.3389/fcimb.2022.811123 35223544 PMC8867070

[27] Cwiklinski K, Robinson MW, Donnelly S, et al. Complementary transcriptomic and proteomic analyses reveal the cellular and molecular processes that drive growth and development of fasciola hepatica in the host liver. BMC Genomics. 2021;22(1):46. doi: 10.1186/s12864-020-07326-y 33430759 PMC7797711

[28] Lopez Corrales J, Cwiklinski K, De Marco Verissimo C, et al. Diagnosis of sheep fasciolosis caused by fasciola hepatica using cathepsin L enzyme-linked immunosorbent assays (ELISA). Veterinary Parasitol. 2021;298:109517. doi: 10.1016/j.vetpar.2021.109517 34271318

[29] Sais D, Chowdhury S, Dalton JP, et al. Both host and parasite non-coding RNAs co-ordinate the regulation of macrophage gene expression to reduce pro-inflammatory immune responses and promote tissue repair pathways during infection with fasciola hepatica. RNA Biol. 2024;21(1):1007–1022. doi: 10.1080/15476286.2024.2408706 PMC11445894 39344634

[30] Panzade G, Li L, Hebbar S, et al. Global profiling and annotation of templated isomiRs dynamics across caenorhabditis elegans development. RNA Biol. 2022;19(1):928–942. doi: 10.1080/15476286.2022.2099646 35848953 PMC9298154

[31] Urgese G, Paciello G, Acquaviva A, et al. isomiR-SEA: an RNA-Seq analysis tool for miRnas/isomiRs expression level profiling and miRNA-mRNA interaction sites evaluation. BMC Bioinformat. 2016;17(1):148. doi: 10.1186/s12859-016-0958-0 PMC4815201 27036505

[32] Lorenz R, Bernhart SH, Honer Zu Siederdissen C, et al. ViennaRNA package 2.0. Algorithms Mol Biol. 2011;6(1):26. doi: 10.1186/1748-7188-6-26 22115189 PMC3319429

[33] Kwon SC, Baek SC, Choi YG, et al. Molecular basis for the single-nucleotide precision of primary microRNA processing. Mol Cell. 2019;73(3):505–518.e5. doi: 10.1016/j.molcel.2018.11.005 30554947

[34] Thornton JE, Du P, Jing L, et al. Selective microRNA uridylation by Zcchc6 (TUT7) and Zcchc11 (TUT4). Nucleic Acids Res. 2014;42(18):11777–11791. doi: 10.1093/nar/gku805 25223788 PMC4191393

[35] Yang A, Shao TJ, bofill-De Ros X, et al. AGO-bound mature miRnas are oligouridylated by TUTs and subsequently degraded by DIS3L2. Nat Commun. 2020;11(1):2765. doi: 10.1038/s41467-020-16533-w 32488030 PMC7265490

[36] Han BW, Hung JH, Weng Z, et al. The 3′-to-5′ exoribonuclease nibbler shapes the 3′ ends of MicroRNAs bound to drosophila Argonaute1. Curr Biol. 2011;21(22):1878–1887. doi: 10.1016/j.cub.2011.09.034 22055293 PMC3236499

[37] Liu N, Abe M, Sabin LR, et al. The exoribonuclease nibbler controls 3′ end processing of MicroRNAs in drosophila. Curr Biol. 2011;21(22):1888–1893. doi: 10.1016/j.cub.2011.10.006 22055292 PMC3255556

[38] Nguyen TL, Nguyen TD, Ngo MK, et al. Noncanonical processing by animal microprocessor. Mol Cell. 2023;83(11):1810–1826.e8. doi: 10.1016/j.molcel.2023.05.004 37267903

[39] Fang W, Bartel DP. The menu of features that define primary MicroRNAs and enable De Novo design of MicroRNA genes. Mol Cell. 2015;60(1):131–145. doi: 10.1016/j.molcel.2015.08.015 26412306 PMC4613790

[40] Auyeung VC, Ulitsky I, McGeary SE, et al. Beyond secondary structure: primary-sequence determinants license pri-miRNA hairpins for processing. Cell. 2013;152(4):844–858. doi: 10.1016/j.cell.2013.01.031 23415231 PMC3707628

[41] Fernandez-Valverde SL, Taft RJ, Mattick JS. Dynamic isomiR regulation in drosophila development. RNA. 2010;16(10):1881–1888. doi: 10.1261/rna.2379610 20805289 PMC2941097

[42] Presslauer C, Tilahun Bizuayehu T, Kopp M, et al. Dynamics of miRNA transcriptome during gonadal development of zebrafish. Sci Rep. 2017;7(1):43850. doi: 10.1038/srep43850 28262836 PMC5338332

[43] Londin E, Loher P, Telonis AG, et al. Analysis of 13 cell types reveals evidence for the expression of numerous novel primate- and tissue-specific microRNAs. Proc Natl Acad Sci USA. 2015;112(10):E1106–15. doi: 10.1073/pnas.1420955112 25713380 PMC4364231

[44] Salem O, Erdem N, Jung J, et al. The highly expressed 5’isomiR of hsa-miR-140-3p contributes to the tumor-suppressive effects of miR-140 by reducing breast cancer proliferation and migration. BMC Genomics. 2016;17(1):566. doi: 10.1186/s12864-016-2869-x 27502506 PMC4977694

[45] van der Kwast R, Woudenberg T, Quax PHA, et al. MicroRNA-411 and its 5′-IsomiR have distinct targets and functions and are differentially regulated in the vasculature under ischemia. Mol Ther. 2020;28(1):157–170. doi: 10.1016/j.ymthe.2019.10.002 31636041 PMC6953895

[46] Burroughs AM, Ando Y, de Hoon MJ, et al. A comprehensive survey of 3′ animal miRNA modification events and a possible role for 3′ adenylation in modulating miRNA targeting effectiveness. Genome Res. 2010;20(10):1398–1410. doi: 10.1101/gr.106054.110 20719920 PMC2945189

[47] Newman MA, Mani V, Hammond SM. Deep sequencing of microRNA precursors reveals extensive 3′ end modification. RNA. 2011;17(10):1795–1803. doi: 10.1261/rna.2713611 21849429 PMC3185913

[48] McCusker P, Hussain W, McVeigh P, et al. RNA interference dynamics in juvenile fasciola hepatica are altered during in vitro growth and development. Int J Parasitol Drugs Drug Resist. 2020;14:46–55. doi: 10.1016/j.ijpddr.2020.08.004 32866764 PMC7475519

[49] bofill-De Ros X, Kasprzak WK, Bhandari Y, et al. Structural differences between pri-miRNA paralogs promote alternative drosha cleavage and expand target repertoires. Cell Rep. 2019;26(2):447–59.e4. doi: 10.1016/j.celrep.2018.12.054 30625327 PMC6369706

[50] Han J, Lee Y, Yeom KH, et al. Molecular basis for the recognition of primary microRNAs by the Drosha-DGCR8 complex. Cell. 2006;125(5):887–901. doi: 10.1016/j.cell.2006.03.043 16751099

[51] Gu S, Jin L, Zhang Y, et al. The loop position of shRnas and pre-miRnas is critical for the accuracy of dicer processing in vivo. Cell. 2012;151(4):900–911. doi: 10.1016/j.cell.2012.09.042 23141545 PMC3499986

[52] Tran N, Ricafrente A, To J, et al. Fasciola hepatica hijacks host macrophage miRNA machinery to modulate early innate immune responses. Sci Rep. 2021;11(1):6712. doi: 10.1038/s41598-021-86125-1 33762636 PMC7990952

[53] Schirle NT, MacRae IJ. The crystal structure of human Argonaute2. Science. 2012;336(6084):1037–1040. doi: 10.1126/science.1221551 22539551 PMC3521581

[54] Elkayam E, Kuhn CD, Tocilj A, et al. The structure of human argonaute-2 in complex with miR-20a. Cell. 2012;150(1):233. doi: 10.1016/j.cell.2012.06.021 PMC3464090 22682761

[55] Smith D, Cwiklinski K, Jewhurst H, et al. An atypical and functionally diverse family of kunitz-type cysteine/serine proteinase inhibitors secreted by the helminth parasite fasciola hepatica. Sci Rep. 2020;10(1):20657. doi: 10.1038/s41598-020-77687-7 33244035 PMC7692546

[56] Czech B, Zhou R, Erlich Y, et al. Hierarchical rules for argonaute loading in drosophila. Mol Cell. 2009;36(3):445–456. doi: 10.1016/j.molcel.2009.09.028 19917252 PMC2795325

[57] Ghildiyal M, Xu J, Seitz H, et al. Sorting of drosophila small silencing RNAs partitions microRNA* strands into the RNA interference pathway. RNA. 2010;16(1):43–56. doi: 10.1261/rna.1972910 19917635 PMC2802036

[58] Mi S, Cai T, Hu Y, et al. Sorting of small RNAs into arabidopsis argonaute complexes is directed by the 5′ terminal nucleotide. Cell. 2008;133(1):116–127. doi: 10.1016/j.cell.2008.02.034 18342361 PMC2981139

[59] Takeda A, Iwasaki S, Watanabe T, et al. The mechanism selecting the guide strand from small RNA duplexes is different among argonaute proteins. Plant And Cell Physiol. 2008;49(4):493–500. doi: 10.1093/pcp/pcn043 18344228

[60] Perez MG, Gillan V, Anderson WM, et al. A secreted helminth microRNA suppresses gastrointestinal cell differentiation required for innate immunity. Front Immunol. 2025;16:1558132. doi: 10.3389/fimmu.2025.1558132 40213548 PMC11983496

[61] Vitkauskaite A, McDermott E, Lalor R, et al. In vitro co-culture of fasciola hepatica newly excysted juveniles (NEJs) with 3D HepG2 spheroids permits novel investigation of host–parasite interactions. Virulence. 2025;16(1):2482159. doi: 10.1080/21505594.2025.2482159 40132201 PMC11938319

