# Supplementary material for: Dynamic shifts in isomiR profiles during parasite maturation of Fasciola hepatica
Source: RNA Biol. 2025 Jul 31;22(1):1–22. doi: 10.1080/15476286.2025.2538271 (PMC12320863; doi:10.1080/15476286.2025.2538271)
Supplement: Data File 1 QC Report.pdf [file KRNB_A_2538271_SM8898.pdf]

1. RNA quality control

Total RNAQuantification and Quality Assuranceby spectrophotometer

Sample Name	OD260/280 Ratio	OD260/230 Ratio	Conc.(ng/μl)	Volume (μl)	Total Amount (ng)
Ad_4	1.82	2.21	469.88	15	7048.2
Ad_5	1.84	2.09	541.66	15	8124.9
Ad_6	1.81	2.16	454.7	15	6820.5
Juv21_1	1.85	2.27	553.78	15	8306.7
Juv21_2	1.84	2.27	558.42	15	8376.3
Juv21_3	1.85	2.19	547.74	15	8216.1
NEJ_1	1.85	1.97	105.97	15	1589.55
NEJ_2	1.83	2.17	122.47	15	1837.05
NEJ_3	1.84	2.01	199	15	2985

*For spectrophotometer, the O.D. A260 /A280 ratio should be close to 2.0 for pure RNA(ratios between 1.8 and 2.1 are acceptable). TheO.D.A260/A230 ratio should be more than 1.8.

RNAIntegrity and gDNAcontamination test by Denaturing AgaroseGel Electrophoresis

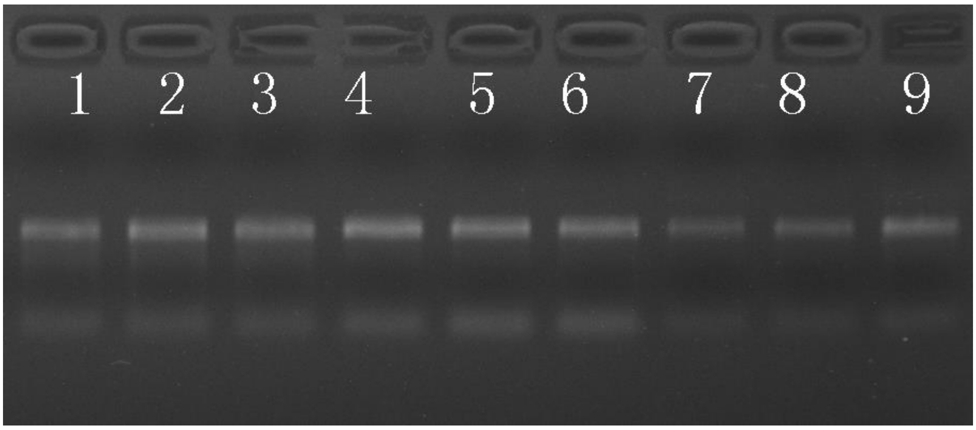

Lane1: Total RNAof sample Ad_4
Lane2: Total RNAof sample Ad_5
Lane3: Total RNAof sample Ad_6
Lane4: Total RNAof sample Juv21_1
Lane5: Total RNAof sample Juv21_2
Lane6: Total RNAof sample Juv21_3
Lane7: Total RNAof sample NEJ_1
Lane8: Total RNAof sample NEJ_2

*The 28S and 18S ribosomal RNA bands should be fairly sharp, intense bands. The intensity of the upper band should be about twice that of the lower band. Smaller, more diffuse bands representing low molecular weight RNAs (tRNA and 5S ribosomal RNA) may be present. It is normal to see a diffuse smear of ethidium bromide staining material migrating between the 18S and 28S ribosomal bands, probably comprised of mRNA and other heterogeneous RNA species. DNA contamination of the RNA preparation will be evident as a high molecular weight smear or band migrating above the 28S ribosomal RNA band. Degradation of the RNA will be reflected by smearing of ribosomal RNA bands.

2. Quality Assessment of Sequencing Library

Sequencing library was determined by Agilent 2100 Bioanalyzer using the Agilent DNA 1000 chip kit (Agilent, part # 5067-1504)

Sample Name	Size (bp)	Conc.(ng/μl)	Conc.(nmol/L)	Volume (μl)	Total Amount (ng)
Ad_4	147	1.75	18.1	20	35
Ad_5	147	1.84	19.0	20	36.8
Ad_6	148	2.12	21.7	20	42.4
Juv21_1	147	1.13	11.7	20	22.6
Juv21_2	147	1.17	12.1	20	23.4
Juv21_3	148	1.84	18.9	20	36.8
NEJ_1	147	0.95	9.8	20	19
NEJ_2	148	0.34	3.5	20	6.8
NEJ_3	147	1.29	13.4	20	25.8

**The libraries were adjusted to 10nM before cluster generation.

Quality control analysis on an Agilent 2100 Bioanalyzer

Sample Ad_4:

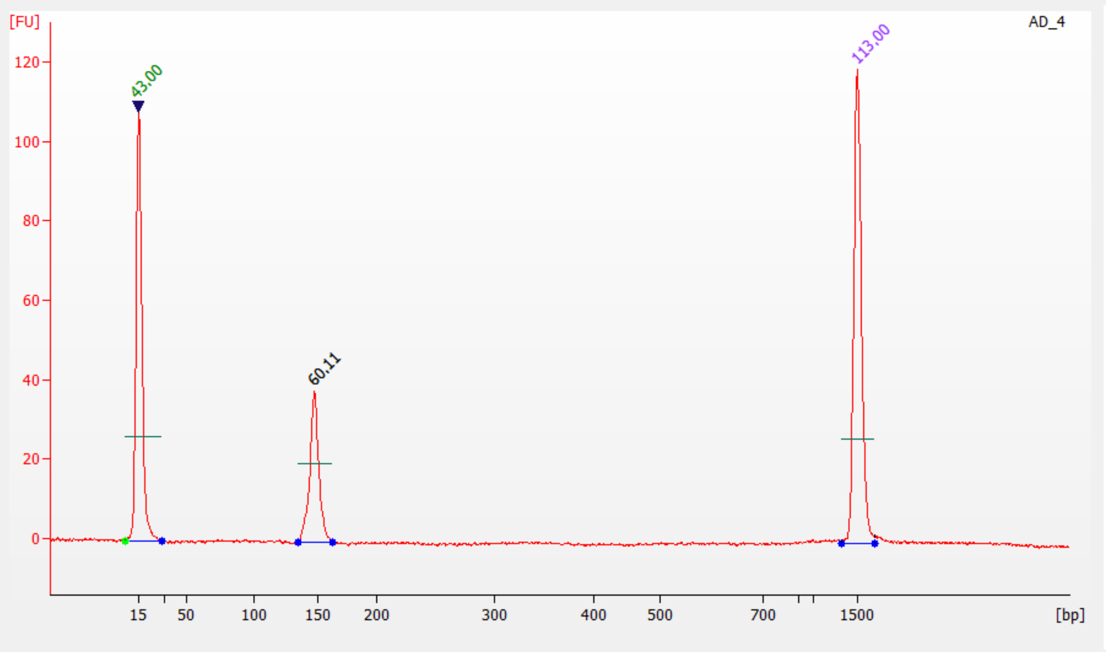

		Size [bp]	Conc. [ng/µl]	Molarity [nmol/l]	Observations
		15	4.20	424.2	Lower Marker
2		147	1.75	18.1	
3		1,500	2.10	2.1	Upper Marker

Sample Ad_5:

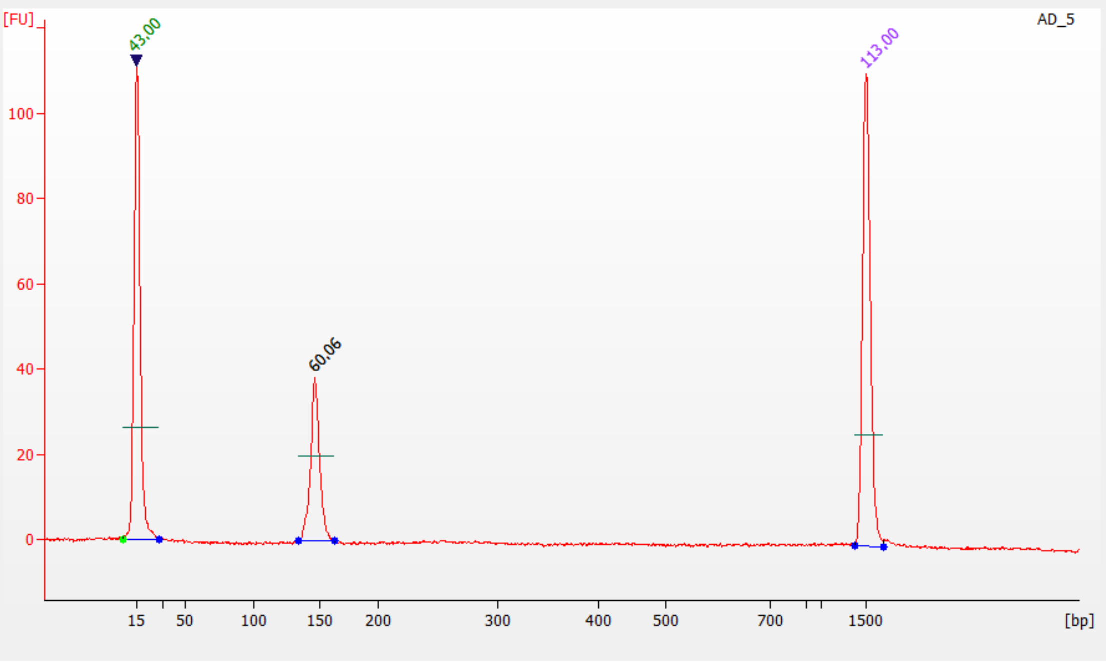

		Size [bp]	Conc. [ng/µl]	Molarity [nmol/l]	Observations
		15	4.20	424.2	Lower Marker
2		147	1.84	19.0	
3		1,500	2.10	2.1	Upper Marker

Sample Ad_6:

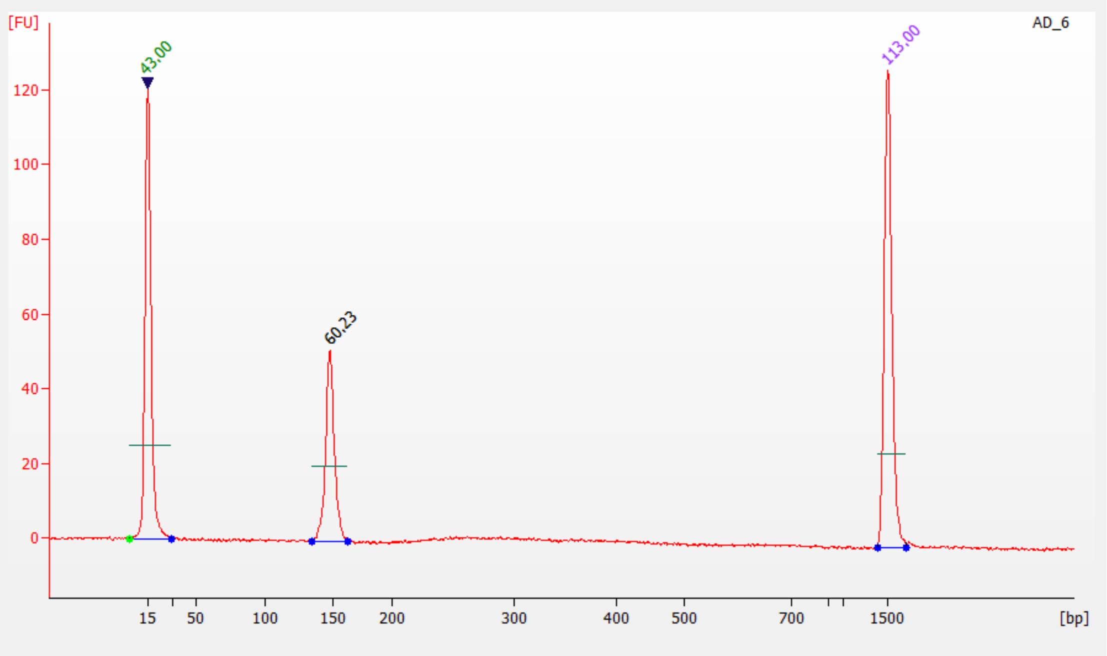

		Size [bp]	Conc. [ng/µl]	Molarity [nmol/l]	Observations
▶	◀	15	4.20	424.2	Lower Marker
2		148	2.12	21.7	
3	▶	1,500	2.10	2.1	Upper Marker

Sample JUV21-1:

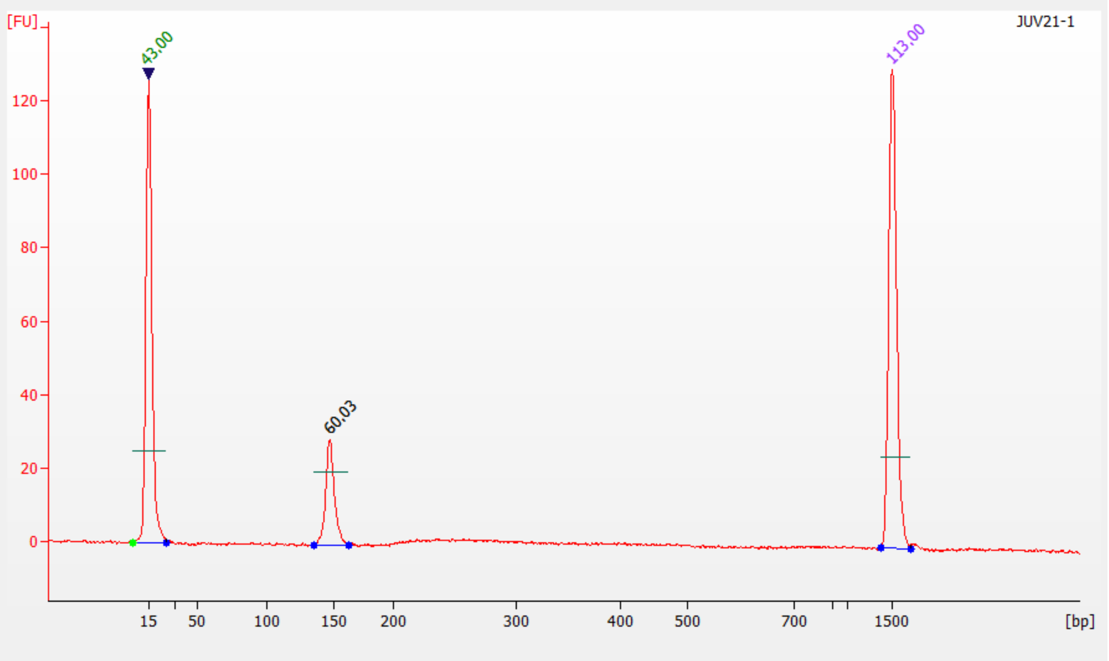

		Size [bp]	Conc. [ng/µl]	Molarity [nmol/l]	Observations
▶	◀	15	4.20	424.2	Lower Marker
2		147	1.13	11.7	
3	▶	1,500	2.10	2.1	Upper Marker

Sample JUV21-2:

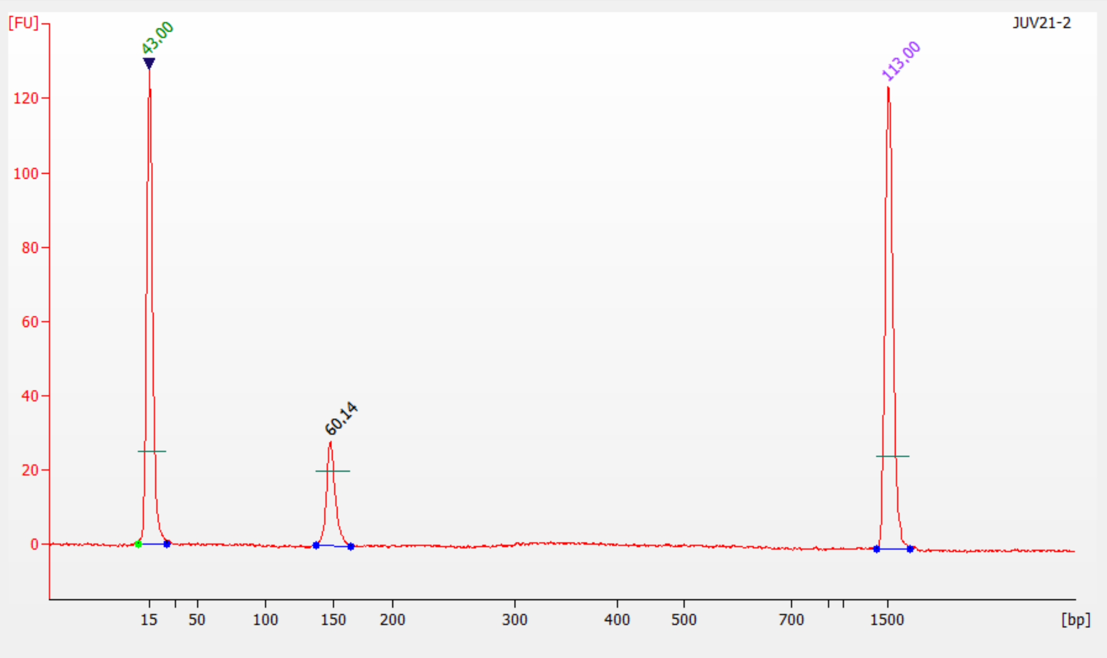

		Size [bp]	Conc. [ng/µl]	Molarity [nmol/l]	Observations
▸	◀	15	4.20	424.2	Lower Marker
2		147	1.17	12.1	
3	▶	1,500	2.10	2.1	Upper Marker

Sample JUV21-3:

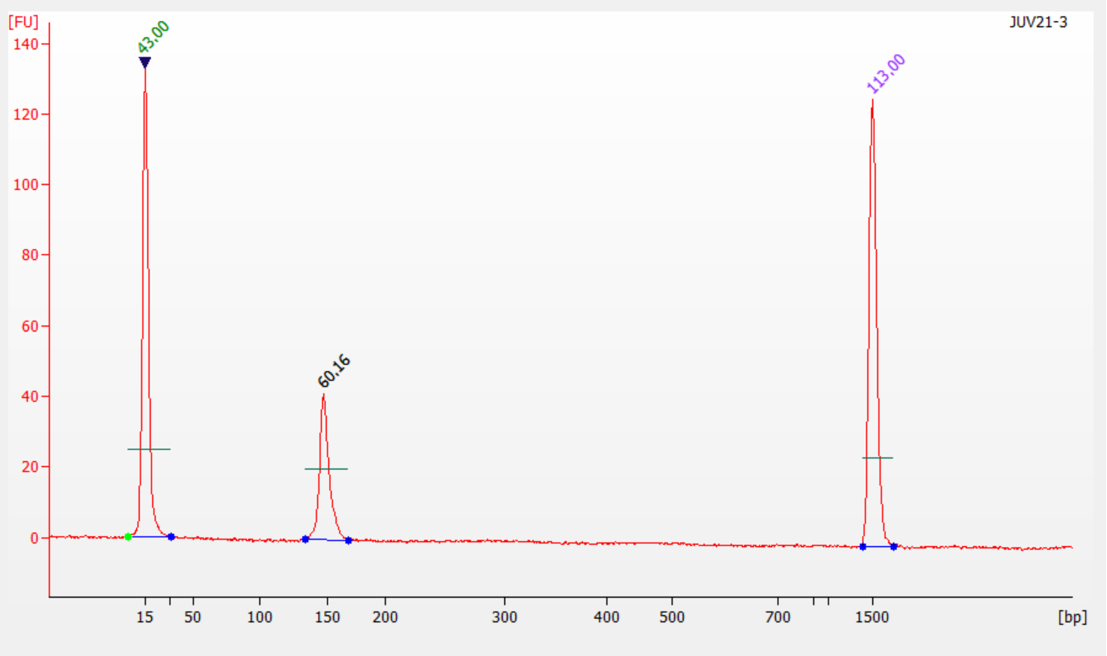

		Size [bp]	Conc. [ng/µl]	Molarity [nmol/l]	Observations
▸	◀	15	4.20	424.2	Lower Marker
2		148	1.84	18.9	
3	▶	1,500	2.10	2.1	Upper Marker

Sample NEJ-1:

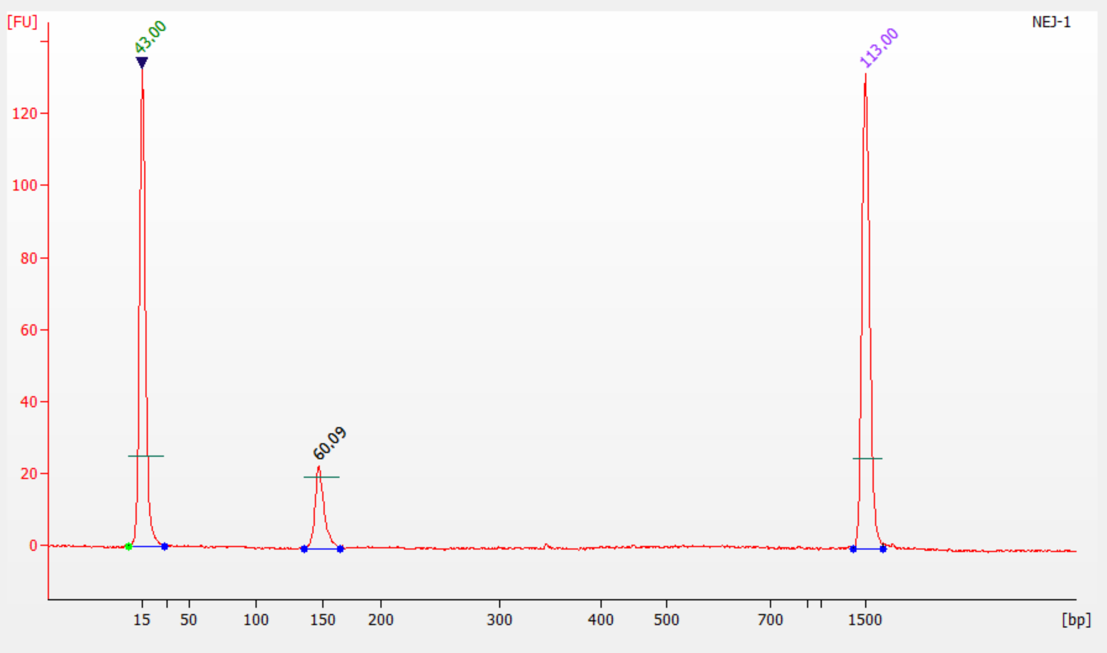

		Size [bp]	Conc. [ng/µl]	Molarity [nmol/l]	Observations
▶	◀	15	4.20	424.2	Lower Marker
2		147	0.95	9.8	
3	▶	1,500	2.10	2.1	Upper Marker

Sample NEJ-2:

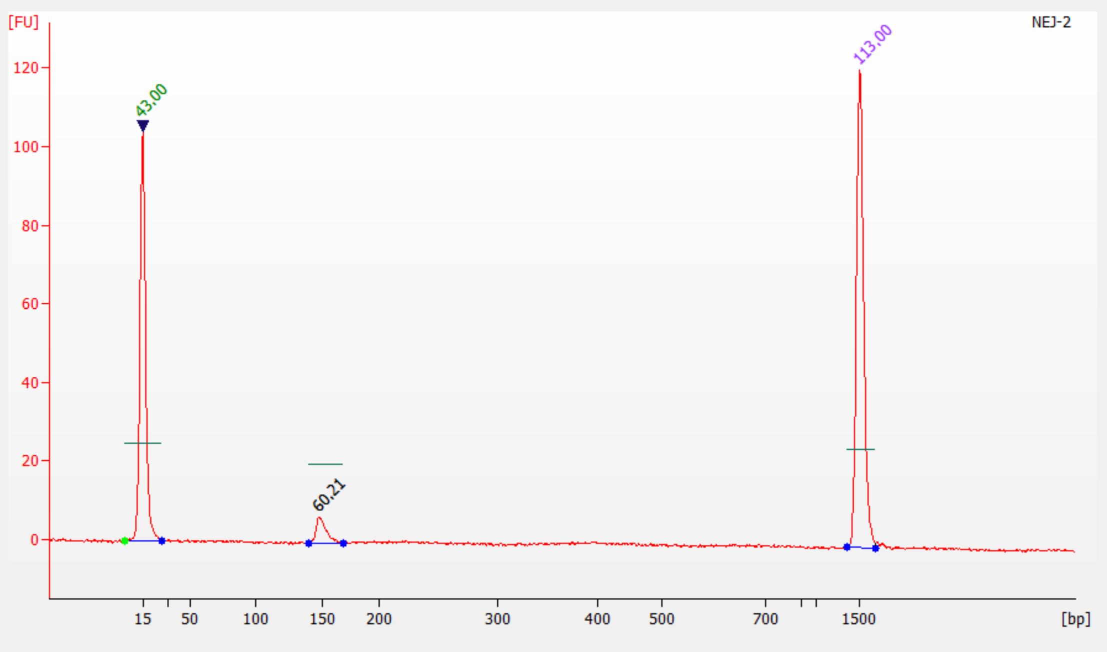

		Size [bp]	Conc. [ng/µl]	Molarity [nmol/l]	Observations
▶	◀	15	4.20	424.2	Lower Marker
2		148	0.34	3.5	
3	▶	1,500	2.10	2.1	Upper Marker

Sample NEJ-3:

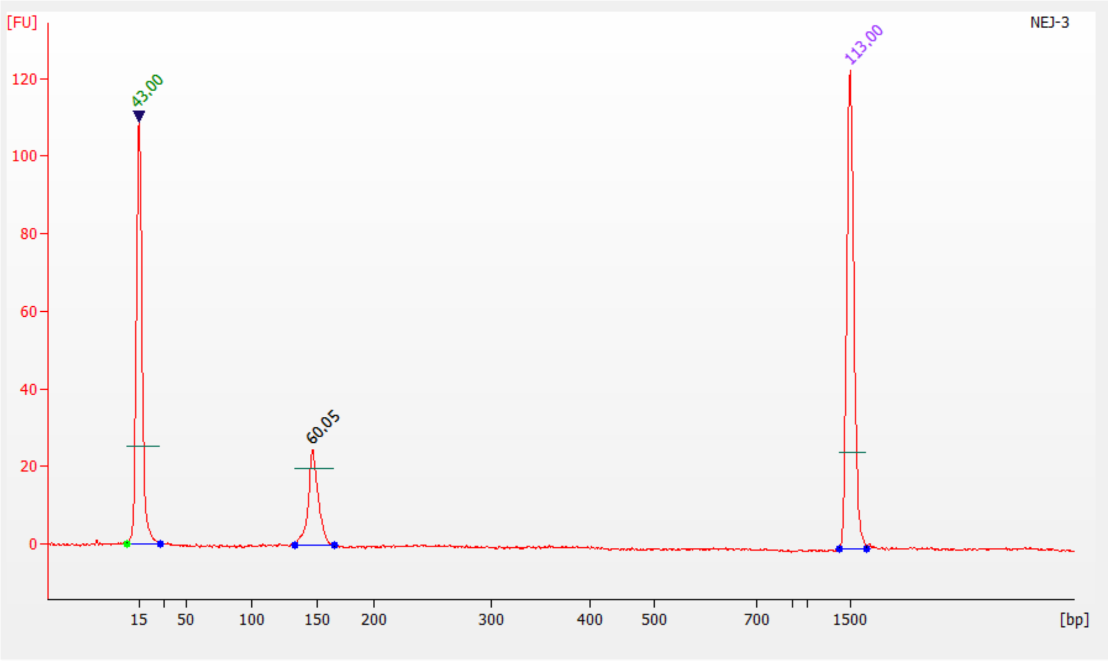

		Size [bp]	Conc. [ng/µl]	Molarity [nmol/l]	Observations
		15	4.20	424.2	Lower Marker
2		147	1.29	13.4	
3		1,500	2.10	2.1	Upper Marker

MultiQC General Statistics

Sample Name	Dups	GC	Avg len	Seqs
Ad_4	64.90%	44.00%	21bp	10.1M
Ad_5	62.90%	44.00%	21bp	11.3M
Ad_6	66.50%	44.00%	21bp	14.0M
Juv21_1	68.20%	43.00%	22bp	10.1M
Juv21_2	69.90%	43.00%	22bp	14.2M
Juv21_3	65.10%	43.00%	22bp	9.8M
NEJ_1	65.70%	42.00%	22bp	7.7M
NEJ_2	67.80%	44.00%	22bp	10.6M
NEJ_3	67.40%	42.00%	22bp	9.9M

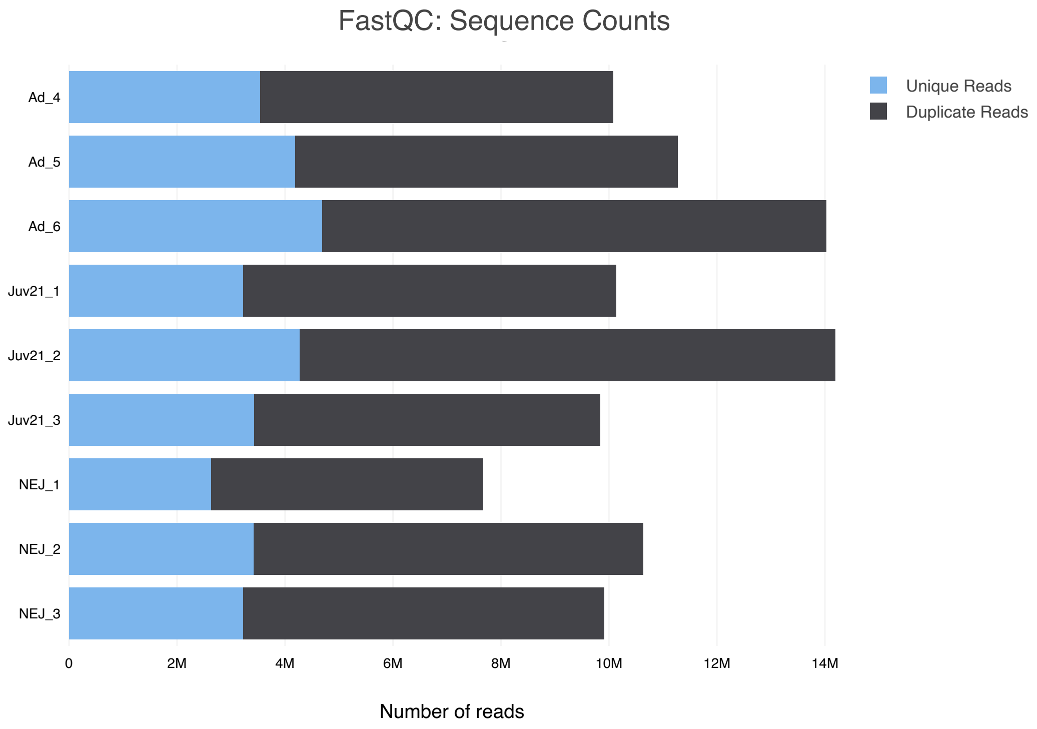

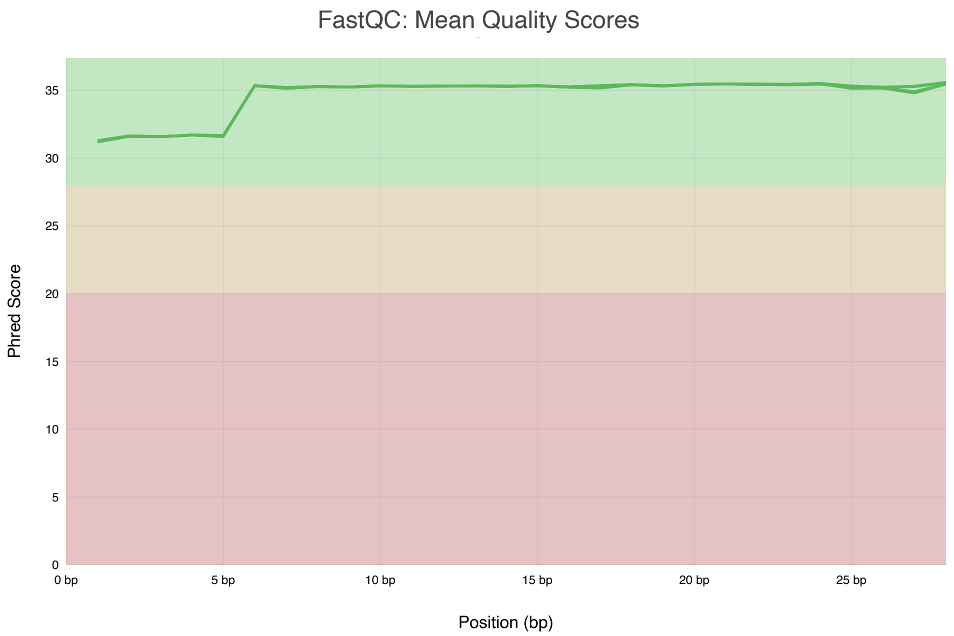

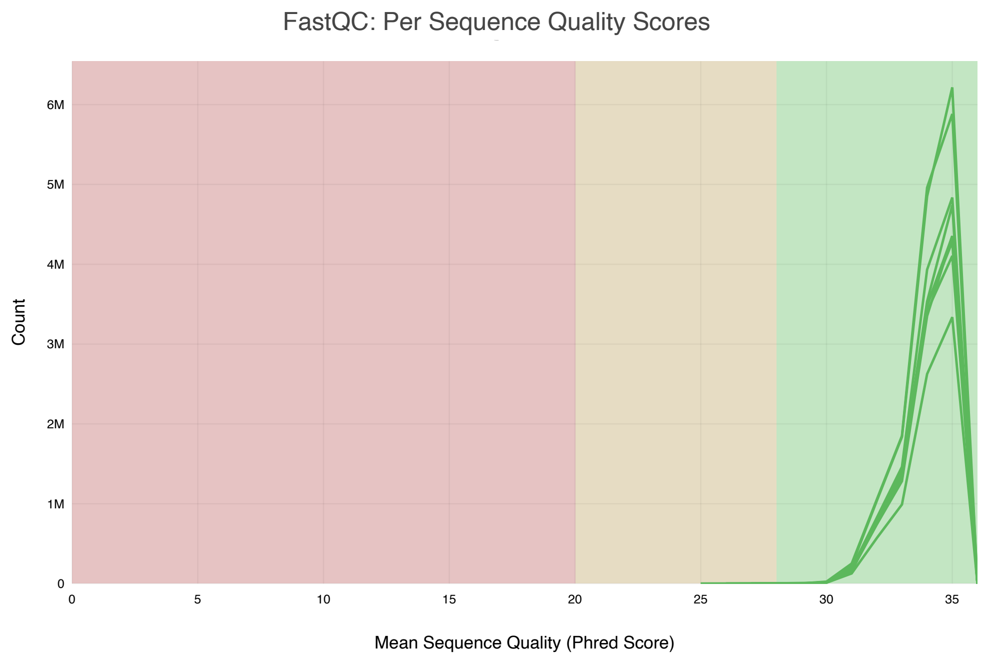
FastQC: Per Sequence Quality Scores
Count
6M
5M
4M
3M
2M
1M
0
0
5
10
15
20
25
30
35
Mean Sequence Quality (Phred Score)

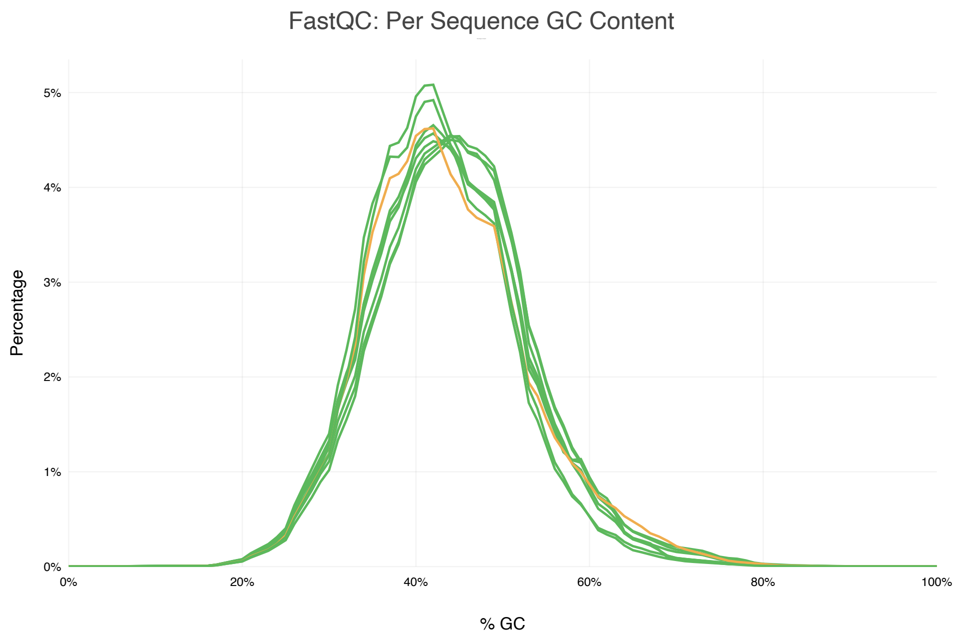
FastQC: Per Sequence GC Content
Percentage
5%
4%
3%
2%
1%
0%
0%
20%
40%
60%
80%
100%
% GC

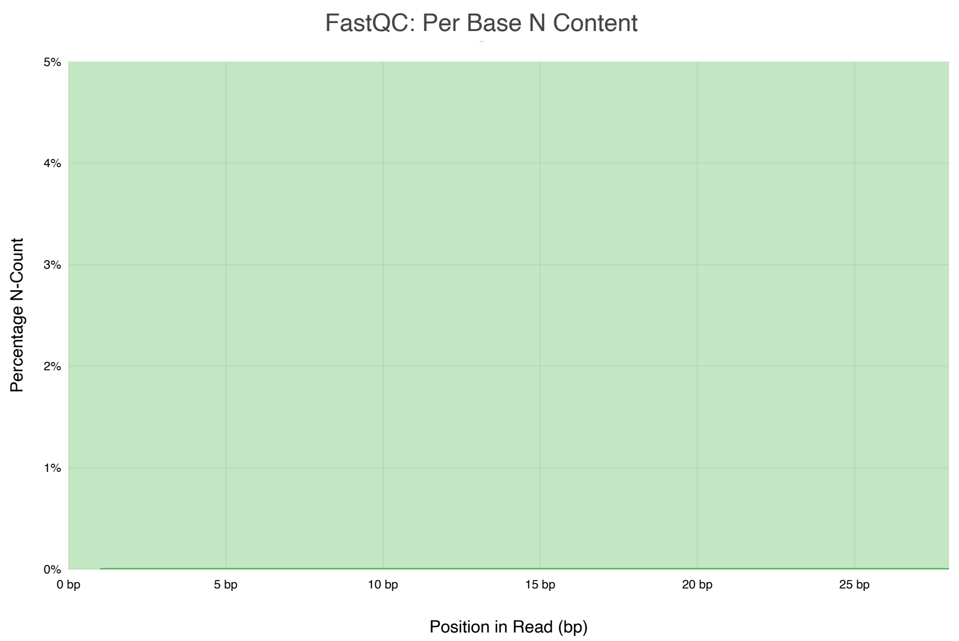
FastQC: Per Base N Content
Percentage N-Count
5%
4%
3%
2%
1%
0%
0 bp
5 bp
10 bp
15 bp
20 bp
25 bp
Position in Read (bp)

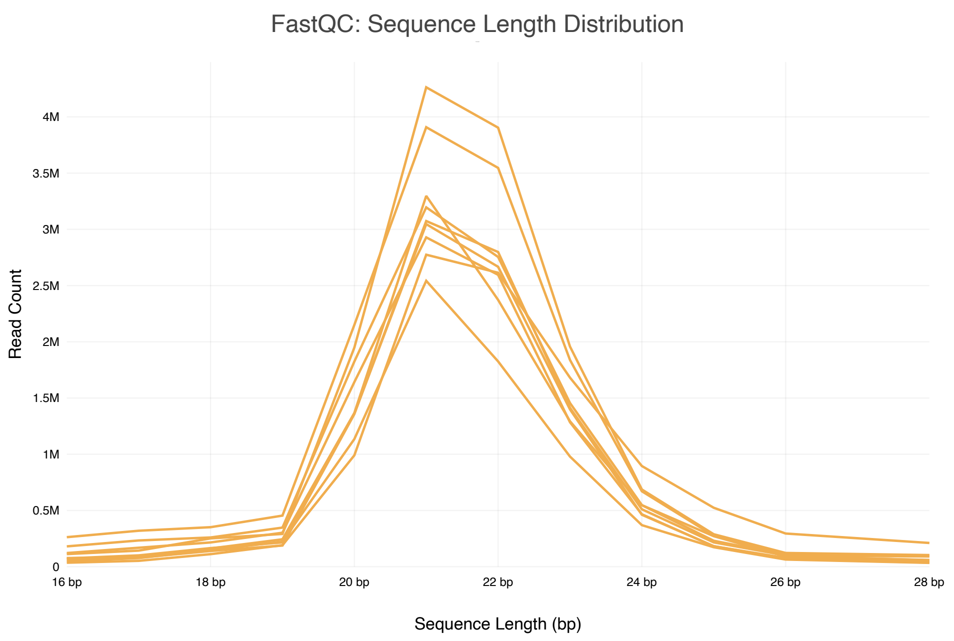
FastQC: Sequence Length Distribution
Read Count
4M
3.5M
3M
2.5M
2M
1.5M
1M
0.5M
0
16 bp
18 bp
20 bp
22 bp
24 bp
26 bp
28 bp
Sequence Length (bp)

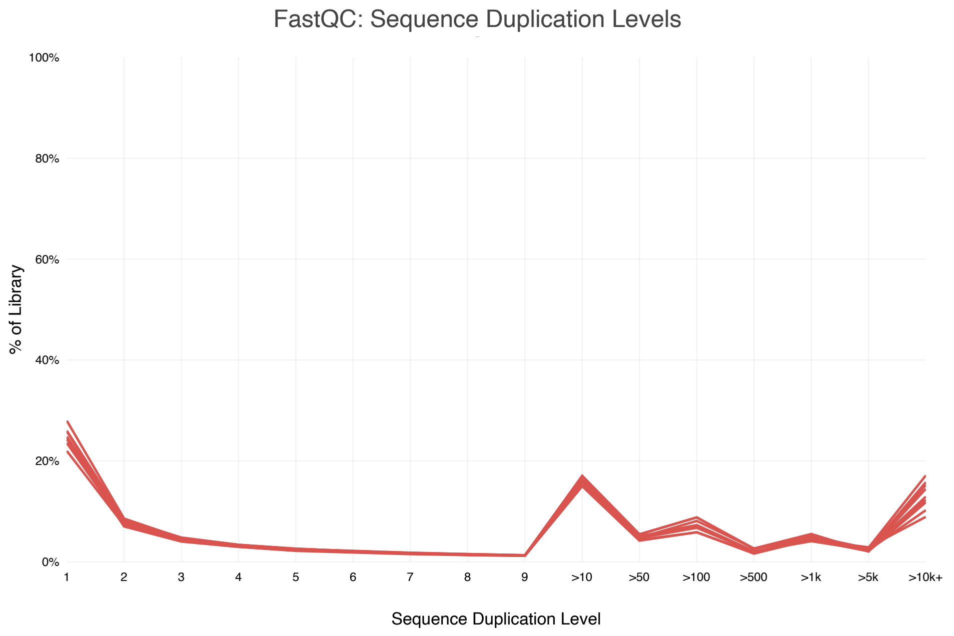
FastQC: Sequence Duplication Levels
% of Library
100%
80%
60%
40%
20%
0%
1
2
3
4
5
6
7
8
9
>10
>50
>100
>500
>1k
>5k
>10k+
Sequence Duplication Level

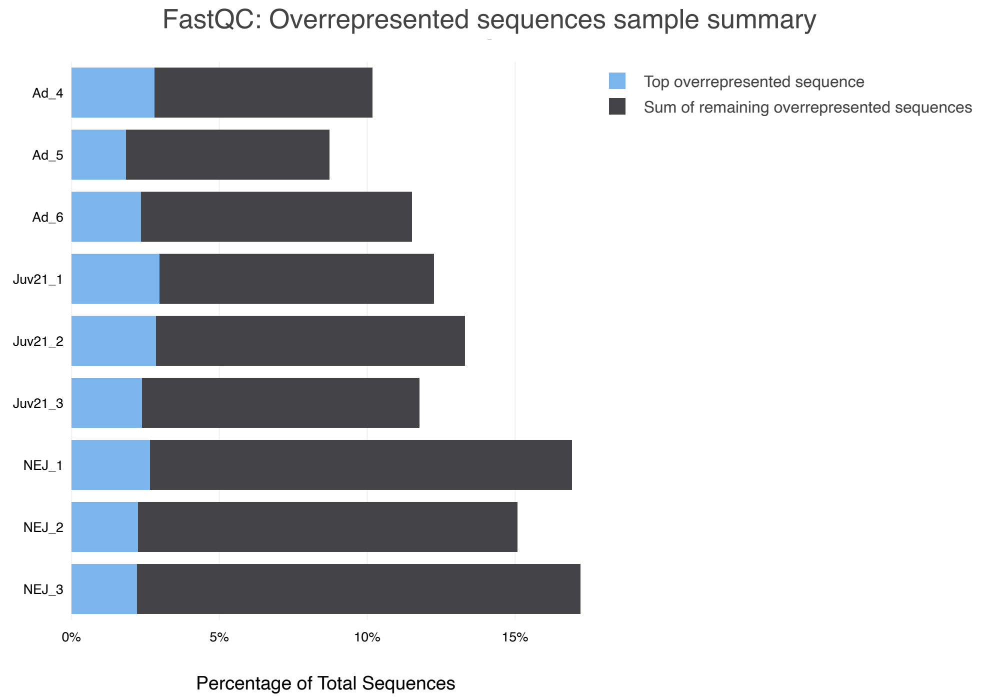
FastQC: Overrepresented sequences sample summary
Top overrepresented sequence
Sum of remaining overrepresented sequences
Ad_4
Ad_5
Ad_6
Juv21_1
Juv21_2
Juv21_3
NEJ_1
NEJ_2
NEJ_3
0%
5%
10%
15%
Percentage of Total Sequences

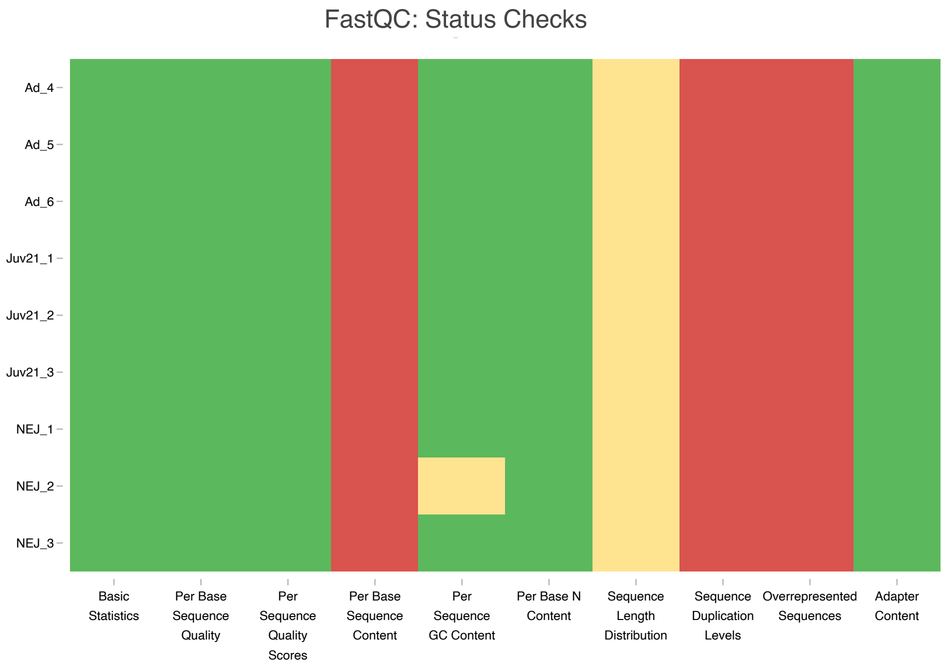
FastQC: Status Checks
Ad_4
Ad_5
Ad_6
Juv21_1
Juv21_2
Juv21_3
NEJ_1
NEJ_2
NEJ_3
Basic Statistics
Per Base Sequence Quality
Per Sequence Quality Scores
Per Base Sequence Content
Per Sequence GC Content
Per Base N Content
Sequence Length Distribution
Sequence Duplication Levels
Overrepresented Sequences
Adapter Content
