# Supplementary figures and images for: Dynamic shifts in isomiR profiles during parasite maturation of Fasciola hepatica
Source: RNA Biol. 2025 Jul 31;22(1):1–22. doi: 10.1080/15476286.2025.2538271 (PMC12320863; doi:10.1080/15476286.2025.2538271)

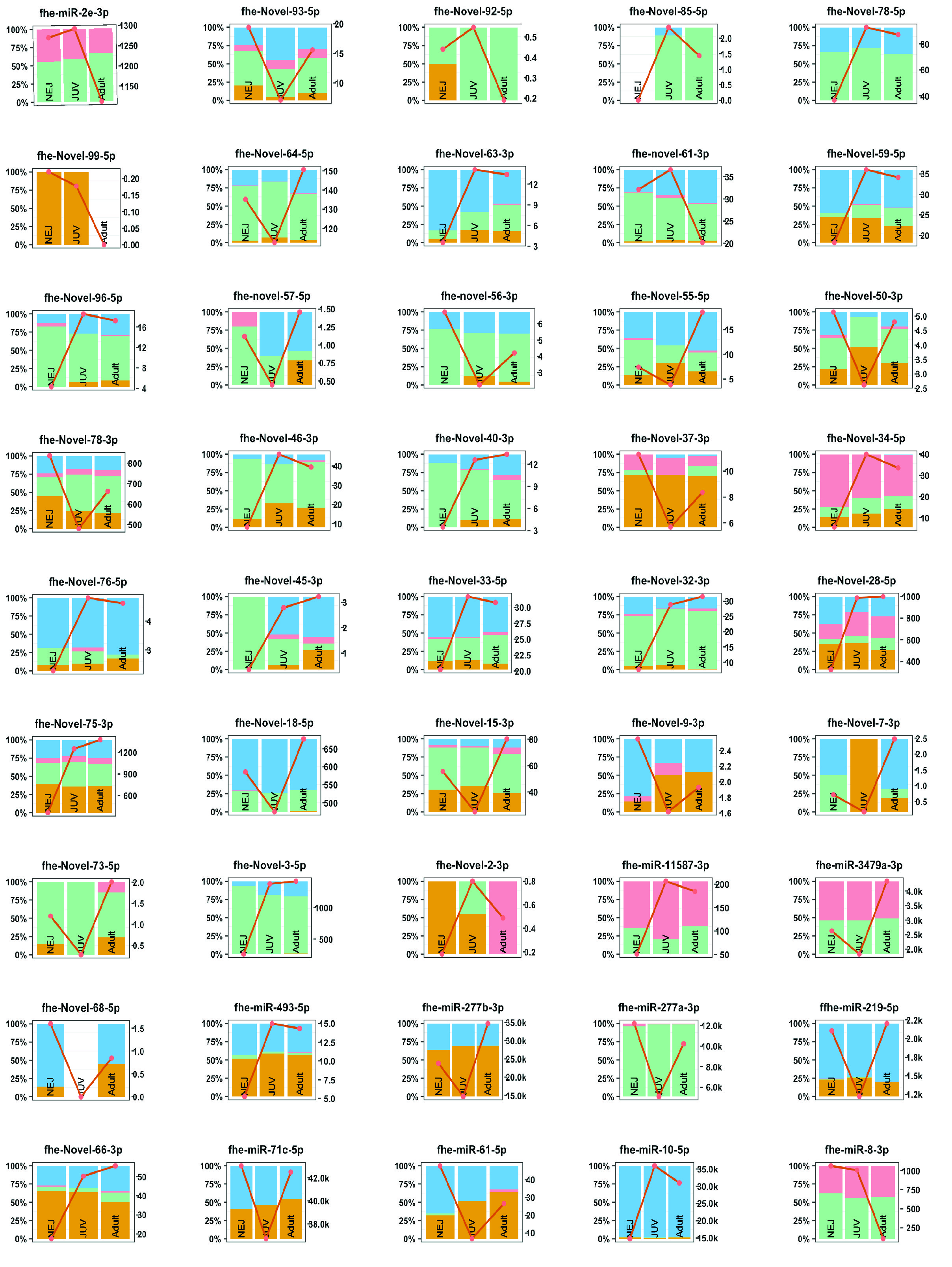

Supplement: Supp Fig 1.jpg [file KRNB_A_2538271_SM8896.jpg]

Supplementary Figure 2

A.

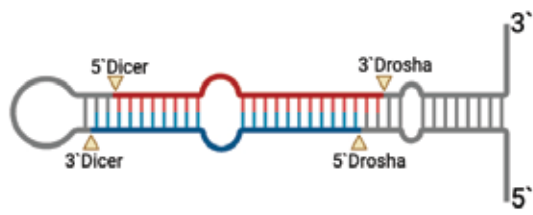

B.

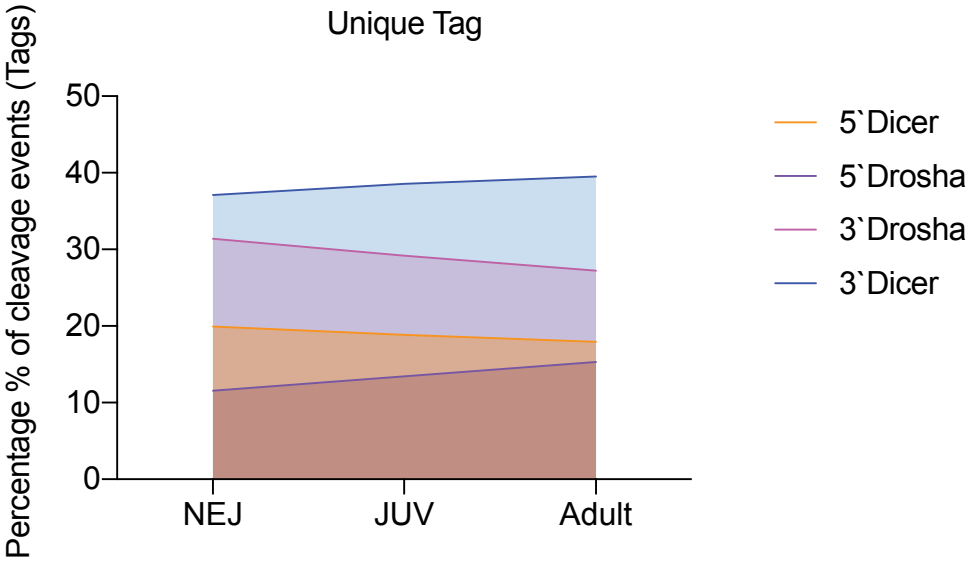

C.

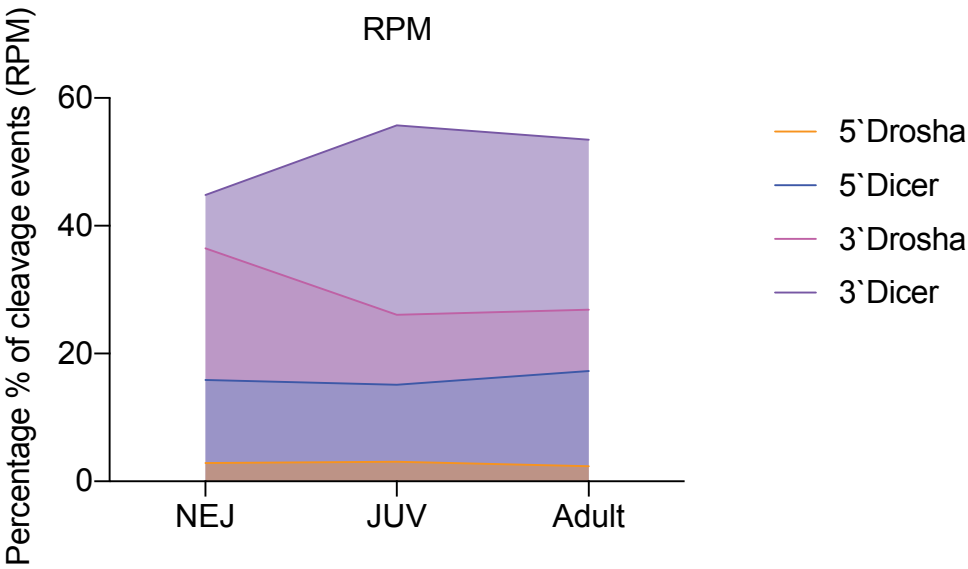

Supplement: Suppl Fig 2.pdf [file KRNB_A_2538271_SM8895.pdf]
